# Customer Experience Management – Eine qualitative Studie zur Umsetzung in der Versicherungsbranche

**DOI:** 10.1007/s12297-022-00531-6

**Published:** 2022-10-20

**Authors:** Lisa-Marie Klopfer, Martina Steul-Fischer, Armin Zitzmann

**Affiliations:** 1 Lehrstuhl für BWL, insb. Versicherungsmarketing, Fachbereich Wirtschafts- und Sozialwissenschaften, Friedrich-Alexander-Universität Erlangen-Nürnberg, Lange Gasse 20, 90403 Nürnberg, Deutschland; 2 NÜRNBERGER Beteiligungs-Aktiengesellschaft, Ostendstraße 100, 90334 Nürnberg, Deutschland

**Keywords:** Customer Experience Management, Kundenzufriedenheit, Kundenzentrierung, Customer experience management, Customer satisfaction, Customer focus

## Abstract

Das Customer Experience Management erfährt in jüngster Zeit sowohl in der Konsumgüter- als auch in der Dienstleistungsbranche erhöhte Aufmerksamkeit. Die Relevanz ergibt sich unter anderem aus dem veränderten Konsumverhalten und den gestiegenen Kundenerwartungen in Folge der Digitalisierung. Auch die Forschung beschäftigt sich zunehmend mit dem Managementansatz, wobei dessen Umsetzung durch Versicherungsunternehmen bisher noch nicht untersucht wurde. Diese Studie beleuchtet daher den Status quo des Customer Experience Managements in der Versicherungsbranche in Form einer qualitativen Studie. Als Fallbeispiel dient die NÜRNBERGER Versicherung, die seit 2016 ein konzernübergreifendes Customer Experience Management für ihre Privatkunden aufbaut. Das Unternehmen hat bereits eine Vielzahl an Maßnahmen ergriffen, um die Bedürfnisse sowie Ansprüche seiner Kunden besser zu verstehen und ihnen somit in jeder Phase der Kundenbeziehung ein optimales Kundenerlebnis zu bieten sowie damit zu einer Verbesserung der Kundenzufriedenheit beizutragen.

## Abstract (en)

Customer experience management has recently received increased attention in both the consumer goods and service industries. The relevance derives in part from the changed consumer behaviour and the increased customer expectations as an outcome of digitalization. Researchers are also increasingly focusing on the management approach, although its implementation by insurance companies has not yet been investigated. This paper therefore examines the status quo of customer experience management in the insurance industry in the form of a qualitative study. NÜRNBERGER Versicherung serves as a case study here. Since 2016 the company has been implementing a customer experience management system for its private customers and has already taken a large number of steps to better understand the needs and demands of its customers in order to offer them an optimal customer experience in every phase of the customer relationship thus contributing to an improvement in customer satisfaction.

## Einführung

Die Digitalisierung und die damit einhergehenden Innovationen im Bereich der Finanztechnologien verändern die Versicherungswirtschaft nachhaltig. Einfachheit, Flexibilität und eine stark ausgeprägte Kundenzentrierung stehen, vor allem aufgrund der digitalen Möglichkeiten, bei vielen Versicherungsunternehmen im Mittelpunkt (Stoeckli et al. 2018). Die Versicherungsnehmer erwarten und honorieren dabei eine einfache, unkomplizierte und transparente Kommunikation sowie individuelle, passgenaue Versicherungslösungen. Im Zuge der Digitalisierung und der Kundenzentrierung haben sich daher die Customer Experience und das Customer Experience Management (CEM) zu zwei höchst relevanten Handlungsfeldern in der Versicherungswirtschaft entwickelt, da wirtschaftliche Erfolge in hohem Maße von der Kundenzufriedenheit dieser Versicherungsnehmer abhängig sind.

Der Grundstein für die heutige Sichtweise der Customer Experience wurde bereits in den 1980er und 1990er-Jahren gelegt. Holbrook und Hirschmann (1982) erweiterten das damalige Verständnis der Konsumforschung, dass Menschen lediglich zur Erfüllung des Konsumbedürfnisses Produkte und Dienstleistungen konsumieren, um die Betrachtungsweise des Konsumerlebnisses als Mittel zur Erfüllung des menschlichen Bedürfnisses nach Erlebnissen. Pine und Gilmore (1998) haben anschließend auf die zunehmende Erlebnisorientierung innerhalb der Gesellschaft und die damit verbundenen Veränderungen der Kundenbedürfnisse aufmerksam gemacht.

Seitdem hat sich die Art und Weise, wie Menschen Produkte und Dienstleistungen konsumieren mehrfach weiterentwickelt, wobei die Digitalisierung eine entscheidende Rolle spielt. Neue Technologien haben nicht nur zu neuen Gestaltungsmöglichkeiten von Erlebnissen im eigentlichen Sinne, wie zum Beispiel interaktiven Virtual Reality Simulationen, beigetragen (Pine und Gilmore 1998). Das Internet und die mobilen Endgeräte haben vor allem zu einer schnellen Ausweitung sowie zu einer Multidimensionalität der verfügbaren Touchpoints und Kommunikationskanäle entlang der Customer Journey eines Konsumenten geführt (Lemon und Verhoef 2016). Die Customer Journey nimmt daher sowohl an Komplexität als auch an Individualität stetig zu (Becker und Jaakkola 2020; Lemon und Verhoef 2016). Den Kunden selbst kann, u. a. durch ihre starke Vernetzung über die sozialen Medien und durch die vielfältigen Möglichkeiten des Informations- und Konsumverhaltens (z. B. Show‑/Webrooming, Online-Reviews, Vergleichsportale), über alle Phasen der Customer Journey hinweg eine deutlich einflussreichere Position als noch vor einigen Jahren zugeschrieben werden. Gleichzeitig lässt sich bei den Kunden eine, durch die Digitalisierung verstärkte, Anspruchsinflation in Bezug auf die Individualität und Schnelligkeit von Services beobachten (Kreutzer 2018).

All diese fortschreitenden Veränderungen stellen Versicherungsunternehmen langfristig vor die Herausforderung, im Rahmen eines ganzheitlichen Managementansatzes die Kundenorientierung in den Mittelpunkt des unternehmerischen Handelns zu rücken und durch eine kontinuierliche Optimierung der Customer Experience einen Mehrwert für den Kunden zu generieren (u. a. Gentile et al. 2007; Prahalad und Ramaswamy 2003). Wem dies gelingt, der kann sich durch eine langfristige Kundenloyalität einen Wettbewerbsvorteil verschaffen – ein Ziel, das in den gesättigten und sich stetig verändernden Märkten von heute nur noch schwer zu erreichen oder dauerhaft zu halten ist (Homburg et al. 2017). Dies ist vor allem in Branchen von Relevanz, in denen die Entwicklung von qualitativ hochwertigen Kundenbeziehungen alleine nicht mehr als Differenzierungsmerkmal angesehen werden kann (Palmer 2010) wie in der Versicherungsbranche.

Neben der enormen Beachtung, die das CEM von Seiten der Unternehmenspraxis erhält, beschäftigt sich seit einiger Zeit auch die Forschung vermehrt mit der Thematik. Insgesamt gilt das Konzept des CEM als nicht ausreichend verstanden, weshalb weiterer und vielfältiger Forschungsbedarf besteht (u. a. Becker und Jaakkola 2020; Homburg et al. 2017; Lemon und Verhoef 2016), beispielsweise hinsichtlich einer fallstudienbasierten Betrachtung des Entwicklungsverlaufs eines CEM im Unternehmen (Jozić 2015). Dieser Artikel beleuchtet daher den Status quo des CEM in der Versicherungsbranche sowie dessen Umsetzung durch die NÜRNBERGER Versicherung auf Basis einer qualitativen Studie. Im folgenden Kapitel werden zunächst die theoretischen und konzeptionellen Grundlagen des CEM erläutert, bevor dessen Relevanz für die Versicherungsbranche aus theoretischer Perspektive dargelegt wird. Einer Vorstellung des methodischen Vorgehens der Studie folgt die Darstellung der Ergebnisse aus den durchgeführten Experteninterviews. Abschließend werden die gewonnenen Erkenntnisse diskutiert.

## Theoretische und konzeptionelle Grundlagen

In einem ersten Schritt wird die Customer Experience als Ausgangsbasis des CEM vorgestellt. Anschließend erfolgt eine Einführung in die theoretischen Grundlagen und die bisherigen Forschungserkenntnisse bezüglich dieses Managementkonzepts. Zuletzt wird die theoretische Relevanz des CEM für die Versicherungsbranche diskutiert.

### Die Customer Experience als Ausgangspunkt des Customer Experience Managements

Das Konstrukt der Customer Experience stellt die inhaltliche Basis für das CEM dar (Homburg et al. 2017). Die Customer Experience kann dabei als multidimensionales Konstrukt beschrieben werden, welches den Fokus auf die kognitiven, emotionalen, verhaltensbezogenen, sensorischen und sozialen Reaktionen sowie Wahrnehmungen eines Kunden hinsichtlich der Kontakte mit einem Unternehmen während der gesamten Customer Journey legt (Homburg et al. 2017; Lemon und Verhoef 2016).

Die Reaktionen der Kunden können sowohl im aktiven, direkten (z. B. Abschluss einer Versicherung) als auch im ungeplanten, indirekten Kontakt (z. B. Word-of-Mouth, Werbung etc.) mit einem Versicherungsunternehmen auftreten (Brakus et al. 2009; Meyer und Schwager 2007), weshalb die Customer Experience durch den Kunden nicht immer bewusst und als solche wahrgenommen wird (Becker und Jaakkola 2020). Der Kontakt mit einem Versicherer, egal in welcher Form, entsteht an einer Vielzahl diverser Online- sowie Offline-Touchpoints, die das Unternehmen nicht immer steuern kann (Kuehnl et al. 2019; Lemon und Verhoef 2016; Palmer 2010; Verhoef et al. 2009).

Die Customer Experience ist ein stark subjektives und kontextspezifisches Konstrukt, da die Reaktionen und Wahrnehmungen eines Kunden sowohl von dessen persönlichen Eigenschaften als auch von situationsbedingten und soziokulturellen Gegebenheiten abhängen (Becker und Jaakkola 2020). Als weitere potenzielle Einflussfaktoren auf die Customer Experience können unter anderem frühere Erlebnisse mit einem Unternehmen, andere Konsumenten, Erwartungen, Einstellungen oder auch gleichzeitig auftretende Erlebnisse im Umfeld des Konsumenten genannt werden (Homburg et al. 2017; Palmer 2010; Puccinelli et al. 2009; Verhoef et al. 2009). Zudem entsteht die Customer Experience, im Vergleich zu vielen anderen Konstrukten der Marketingforschung, nicht erst nach der Inanspruchnahme einer Dienstleistung, sondern kann im Rahmen verschiedener, einzelner Interaktionen über alle Phasen der Customer Journey (d. h. Vorkauf‑, Kauf- und Nachkaufphase) hinweg auftreten und als ganzheitliches Erlebnis wirken (Brakus et al. 2009; Puccinelli et al. 2009, Verhoef et al. 2009). Aus diesem Grund ist es wichtig, die Customer Experience als holistisches Konstrukt zu verstehen (Brakus et al. 2009; Lemon und Verhoef 2016; Verhoef et al. 2009).

Becker und Jaakkola (2020) stellen in ihrer aktuellen Studie rund um den Stand der Forschung zu Customer Experience die kritische Frage, ob Unternehmen die Customer Experience überhaupt managen können. Die Autorinnen argumentieren, dass Unternehmen weder die Customer Experience eines Konsumenten kreieren noch die subjektiven Reaktionen der Kunden auf die Customer Experience steuern können (Becker und Jaakkola 2020). Jedoch sei es möglich, eine Vielzahl an Stimuli, die die Customer Experience wiederum beeinflussen, zu überwachen, zu gestalten und zu managen (Becker und Jaakkola 2020).

### Customer Experience Management

Das CEM stellt einen strategischen Managementprozess dar, der die optimale Gestaltung der gesamten Customer Experience eines Kunden mit dem Produkt oder dem Unternehmen an sämtlichen Touchpoints anstrebt (Schmitt 2003). Homburg et al. (2017) sehen in diesem Managementansatz eine übergeordnete Unternehmensressource, die drei wichtige Kernkategorien umfasst: eine auf die Customer Experience gerichtete kulturelle Orientierung des Unternehmens, eine strategische Ausrichtung hinsichtlich der Gestaltung von Customer Experiences sowie die unternehmerischen Fähigkeiten zur kontinuierlichen Erneuerung und Verbesserung der Customer Experience. Die erfolgreiche und effektive Implementierung eines CEM bedarf der unternehmensweiten Umsetzung und einem ganzheitlichen Zusammenspiel der genannten Kategorien (Homburg et al. 2017). Ein wichtiges Ziel des CEM stellt dabei die Wertschaffung für Kunden sowie Unternehmen und damit das Erreichen einer langfristigen Kundenloyalität als auch eines nachhaltigen Wachstums dar (Grewal et al. 2009; Homburg et al. 2017). Daher sollte neben der Schaffung positiver Customer Experiences auch die aktive Vermeidung negativer Customer Experiences in den Managementansatz einbezogen werden.

Generell besteht die wissenschaftliche Auseinandersetzung mit dem CEM mehrheitlich aus theoretischen und konzeptionellen Arbeiten (siehe Tab. 1), während empirische Untersuchungen selten zu finden sind (Lemon und Verhoef 2016). Dies liegt darin begründet, dass keine Skala zur vollumfänglichen Messung der Customer Experience sowie zur Anwendung für alle Branchen und Kontaktkanäle vorhanden ist und sich die Forschung derzeit in der Skalenentwicklung befindet (Becker und Jaakkola 2020; Lemon und Verhoef 2016). Bisher bestehen lediglich erste Ansätze wie beispielsweise zur Messung der Brand Experience (Brakus et al. 2009), der Customer Experience Quality (u. a. Klaus und Maklan 2012) oder der Effektivität des Customer Journey Designs (Kuehnl et al. 2019). Auch in Bezug auf Kenngrößen des Erfolgs eines CEM besteht in der Forschung kein Konsens. Auf Basis des CEM Konzepts von Homburg et al. (2017) entwickeln Klink et al. (2021) eine Skala zur Messung der CEM Reife in einem Unternehmen und stellen anschließend in ihrer quantitativen Studie einen positiven Zusammenhang zwischen dem CEM eines Unternehmens und dessen finanzieller Performance fest. Jedoch wurde die finanzielle Performance anhand der Wahrnehmung der Studienteilnehmer auf einer Skala und nicht objektiv gemessen (Klink et al. 2021). Weiterhin fällt auf, dass der inhaltliche Fokus der Arbeiten zumeist auf der Customer Experience sowie einzelner Aspekte dieser liegt und der ganzheitliche Managementansatz nur in wenigen Studien ausführlich betrachtet wird. Mit der Fragestellung einer unternehmensinternen Implementierung eines CEM beschäftigen sich dagegen Mayer-Vorfelder (2012a) und Homburg et al. (2017) eingehend. Das Konzept eines CEM im Dienstleistungsbereich strukturiert Mayer-Vorfelder (2012a) anhand der idealtypischen Phasen eines entscheidungsorientierten Managementprozesses in Analyse, Planung, Umsetzung sowie Kontrolle und stellt für jeden Prozessschritt entsprechend theoriebasierte Verfahren und Methoden vor (z. B. Analysephase: Kontaktpunkt-Identifikation durch Blueprinting, Kontaktpunkt-Erlebnismessung durch Critical Incident Technique). Das auf Basis einer Grounded Theory-Studie entstandene Framework des CEM nach Homburg et al. (2017) untergliedert die drei Kernkategorien des CEM in jeweils drei bis vier Unterkategorien (z. B. unternehmerische Fähigkeiten im Touchpoint Journey Design, Touchpoint Priorisierung, Touchpoint Journey Monitoring und Touchpoint Adaption).

**Table 1:** 

Autor(en)	Untersuchungsschwerpunkt(e)	Methodik	Branche(n)
Brakus et al. (2009)	Konzeptualisierung der Brand Experience (BE) Entwicklung einer Skala zur Messung der BE Aufdecken eines direkten Einflusses der BE auf Kundenzufriedenheit und -loyalität; indirekter Einfluss über Markenpersönlichkeit	Quantitativ Einfluss BE auf Kundenzufriedenheit und -loyalität	Diverse Konsumgüter- und Dienstleistungsmarken
Grewal et al. (2009)	Entwicklung eines Frameworks zur CE im Einzelhandel Rolle von Makrofaktoren und vom Unternehmen kontrollierten Faktoren für die CE und wichtige Unternehmenskennzahlen	Konzeptionell	Einzelhandel
Homburg et al. (2017)	Konzeptualisierung und Abgrenzung des CEM als Marketingkonzept Ausgestaltung des CEM in der Praxis Entwicklung einer Typologie mit vier CEM-Mustern basierend auf Unternehmensgröße und Kontinuität der Austauschbeziehung	Grounded Theory-Studie inkl. Literaturanalyse und 52 Tiefeninterviews	Diverse Konsumgüter- und Dienstleistungsbereiche
Klink et al. (2021)	Entwicklung einer Skala zur Operationalisierung des CEM auf Basis des Konzepts von Homburg et al. (2017) Aufdecken eines positiven Zusammenhangs zwischen CEM und finanzieller Unternehmensperformance; Marktturbulenzen, Wettbewerbsintensität und technologische Turbulenzen als Moderatoren	Quantitativ	Diverse Konsumgüter- und Dienstleistungsbereiche
Lemon und Verhoef (2016)	Definitionen, Konzepte und Ursprünge der Forschung zu CE im Marketing Forschungsüberblick zu CE, Customer Journeys und CEM Kritische Bereiche für zukünftige Forschung	Literaturüberblick	Branchenübergreifend
Mayer-Vorfelder (2012a)	Konzeption eines systematischen CEM im Dienstleistungsbereich Managementprozess des CEM untergliedert in die Phasen Analyse, Planung, Umsetzung, Kontrolle	Konzeptionell	Dienstleistungen
Meyer und Schwager (2007)	Differenzierung CEM vs. CRM Methoden der Messung, Analyse und Nutzung von Informationen über die CE als Teil eines funktionsübergreifenden CEM Systems	Konzeptionell	Branchenübergreifend
Palmer (2010)	Entwicklung eines konzeptionellen Frameworks der CE, das zwischenmenschliche Beziehungen, Servicequalität und Marken integriert Diskussion bestehender Ansätze der Definition, Theoriebildung und Messung der CE sowie Gründe für das wachsende Interesse an CEM	Literaturüberblick	Branchenübergreifend
Puccinelli et al. (2009)	Theorien und Forschungsrichtungen des Konsumentenverhaltens mit Bezug zu Gestaltungs- und Einflussfaktoren der CE Diskussion der Rolle dieser Faktoren anhand der Phasen des Entscheidungsprozesses des Konsumenten	Literaturüberblick	Einzelhandel
Verhoef et al. (2009)	Entwicklung eines theoriebasierten konzeptionellen Frameworks der Kreation einer CE (Determinanten und Moderatoren der CE) Diskussion zukünftiger Forschungsfragen, u. a. zu CEM Strategien	Literaturüberblick	Einzelhandel

*CE* Customer Experience, *CEM* Customer Experience Management, *CRM* Customer Relationship Management

Des Weiteren betrachten viele Studien das Forschungsgebiet allgemein bzw. branchenübergreifend oder legen den Schwerpunkt auf die Konsumgüterbranche, während Dienstleistungen nur selten im Fokus stehen. Für Dienstleistungen wie Versicherungen hat das CEM ebenso eine hohe Relevanz, welche im Folgenden kurz aus theoretischer Perspektive dargelegt und im weiteren Verlauf der Studie durch die Praxissicht angereichert wird.

### Relevanz des Customer Experience Managements für die Versicherungsbranche

Die frühe Forschung zum Management der Customer Experience legte den Fokus bereits auf Dienstleistungen und setzte beispielsweise bei dem Service Experience Design an (u. a. Edvardsson et al. 2005; Patrício et al. 2011; Zomerdijk und Voss 2010). Aufgrund ihrer konstitutiven Merkmale (u. a. Intangibilität der Leistung, Heterogenität der Leistungserbringung; Parasuraman et al. 1985) zeichnen sich Dienstleistungen im Vergleich zu Konsumgütern allgemein durch eine höhere wahrgenommene Komplexität, Entscheidungsunsicherheit und ein gesteigertes Kaufrisiko aus (Zeithaml 1981). Für eine Qualitätsbeurteilung wird daher meist der Prozess der Leistungserbringung herangezogen, wobei Kunden im Rahmen einer Dienstleistung häufig mehr Touchpoints mit einem Unternehmen und dessen Servicepersonal haben als bei dem Kauf von Konsumgütern (Berry et al. 2006; Kuehnl et al. 2019; Parasuraman et al. 1985). In Bezug auf die Customer Experience im Dienstleistungsbereich schlussfolgern Kuehnl et al. (2019) daher, dass ein effektives Customer Journey Design (z. B. konsistentes Auftreten des Personals über alle Touchpoints) von höherer Wichtigkeit im Vergleich zur Konsumgüterbranche sein kann, um durch dessen Reduktion der wahrgenommenen Komplexität und Unsicherheit zu einer positiven Markeneinstellung sowie Kundenloyalität beizutragen. In der Versicherungswirtschaft kann jedoch beispielsweise ein konsistenter und kundenorientierter Auftritt über alle Vertriebskanäle einerseits aufgrund der mit der Vertriebsstruktur (Direktvertrieb vs. Service-Versicherer) einhergehenden Vielseitigkeit der Touchpoints (z. B. Vertriebspartner nicht immer vollends steuerbar) und andererseits durch häufig uneinheitliche Kundeninformationen infolge des Spartentrennungsgebots erschwert werden (Theis und Wiener 2018).

Versicherungsunternehmen befinden sich in einem stark umkämpften Markt mit einer hohen Regulierung, dessen Kunden bei der Auswahl von Versicherungsprodukten höchsten Wert auf ein angemessenes Preis-Leistungs-Verhältnis legen und diese dadurch zunehmend austauschbar erscheinen lassen (Theis und Wiener 2018). Daher müssen die Unternehmen über den kompetenten Service hinaus qualitativ hochwertige Kundenerlebnisse bieten, um sich von der Konkurrenz abzuheben (Gao et al. 2020; Klaus und Maklan 2012). Dies ist jedoch nicht für alle Dienstleistungsformen gleich einfach umzusetzen. Im Vergleich zu hedonistischen Dienstleistungen (z. B. Konzertbesuch), bei denen das Erlebnis selbst im Mittelpunkt steht, stellt die Customer Experience bei utilitaristischen Dienstleistungen, wie es Versicherungen oder Dienstleistungen des täglichen Bedarfs sind, in der Regel nicht die Kernleistung dar (Gilovich und Gallo 2020; Mayer-Vorfelder 2012b; Van Boven und Gilovich 2003). Nichtsdestotrotz können auch Versicherungskunden eine außergewöhnliche Customer Experience erleben, da sich diese auf die Reaktionen eines jeden einzelnen Kunden auf diverse Stimuli bezieht und nicht zwangsläufig auf die angebotene utilitaristische Dienstleistung selbst (Becker und Jaakkola 2020). Die Herausforderung für Versicherungsunternehmen besteht also darin, möglichst alle Leistungen so zu erbringen, dass sie von dem Kunden als besonderes und positives Erlebnis wahrgenommen werden (Mayer-Vorfelder 2012b). Hierbei kann das CEM einen wertvollen und entscheidenden Beitrag leisten. Welcher Stellenwert dem Managementansatz aus Sicht der Versicherungspraxis zugeschrieben wird und wie dessen Umsetzung aussehen kann, wurde anhand einer qualitativen Studie näher untersucht.

## Qualitative Studie zum Customer Experience Management in der Versicherungsbranche

Aufgrund der geringen Anzahl an bisherigen Forschungsarbeiten zu CEM (Cepeda und Martin 2005; Homburg et al. 2017) und dem frühen Stadium der Forschung hinsichtlich des Managementansatzes in der Versicherungswirtschaft (Roethlisberger 1977 zitiert nach Cepeda und Martin 2005), untersucht diese Studie das Forschungsfeld auf explorative Weise über eine Fallstudie (Yin 2009). Diese Methodik ermöglicht die Beantwortung grundsätzlicher Fragestellungen nach dem ‚Warum?‘, in Bezug auf den Stellenwert des Managementansatzes für Versicherer, und dem ‚Wie?‘, hinsichtlich der ganzheitlichen Umsetzung des CEM in einem Versicherungsunternehmen (Cepeda und Martin 2005; Yin 2009). In dieser Studie wurde sich für die Betrachtung eines Unternehmens entschieden, um durch eine detailliertere Untersuchung und Darstellung der Implementierung des CEM ein tiefgreifendes Verständnis für den Untersuchungsgegenstand zu erzielen (Dyer und Wilkins 1991; Yin 2009). Als Fallbeispiel dient dabei die NÜRNBERGER Versicherung, die seit 2016 ein konzernübergreifendes CEM für ihre Privatkunden aufbaut und somit den Implementierungsprozess bereits durchlaufen hat sowie erste Erfolge erzielen konnte.

### Zielsetzung und Aufbau der Studie

Ziel ist es, einen praxisnahen Einblick in die Entwicklung und konzernübergreifende Implementierung des CEM der NÜRNBERGER Versicherung zu gewähren. Die vorangegangene Literaturrecherche bildet die Basis und der Managementprozess des CEM nach Mayer-Vorfelder (2012a) den konzeptionellen Rahmen für die qualitative Studie in Form von Experteninterviews (Cepeda und Martin 2005). Die Struktur der Studie basiert daher auf dem Verständnis des CEM als datenbasierter und entscheidungsorientierter Managementprozess, der sich in der Aufbau- und Implementierungsphase typischerweise in die Teilbereiche Analyse, Planung, Umsetzung und Kontrolle gliedert (Mayer-Vorfelder 2012a). Es wurden Fragestellungen zu verschiedenen Themenschwerpunkten innerhalb dieser Teilbereiche (Mayer-Vorfelder 2012a) erstellt (z. B. strukturelle, systemseitige und kulturelle Umsetzung) und um Einstiegsfragen sowie Fragen zum Ausblick hinsichtlich des CEM ergänzt. Da die Experten an und in verschiedenen Schnittstellen und Phasen des CEM beteiligt sind, wurden die Fragestellungen der Studie je nach fachlicher Spezialisierung auf die Interviewpartner angepasst. Der Interviewleitfaden ähnelte dabei einem variablen Baukastensystem, d. h. nicht jedes Interview enthielt Fragen aus allen Teilbereichen. Die Einstiegsfragen sowie die Fragen zum Ausblick wurden von allen Experten beantwortet.

### Datenerhebung und Datenauswertung

Die zehn Experten dieser Studie sind aufgrund ihres spezifischen und vielfältigen Praxis- und Erfahrungswissens in Bezug auf das CX-Management^1^ der NÜRNBERGER Versicherung rekrutiert worden (Bogner et al. 2014). Einen Einblick in das CX-Management des Unternehmens aus einer übergeordneten Perspektive ermöglichte die Befragung eines Vorstandsmitglieds der NÜRNBERGER Versicherung, der Projektleitung für die Entwicklung und Implementierung des CX-Managements der NÜRNBERGER Versicherung sowie des Geschäftsführers und der Senior Consultant der MSR Consulting Group, die den initialen Aufbau des CX-Managements im Unternehmen begleitet haben. Interviews mit drei CX-Managern sowie drei Kundenlotsen, die in ihrer täglichen Arbeit das CEM im Unternehmen vorantreiben, lieferten zusätzlich detaillierte Einblicke in die operative Umsetzung des Managementansatzes.

Die qualitative Studie wurde zwischen November 2020 und Februar 2021 in einem zweistufigen Prozess durchgeführt. Zunächst erhielten alle Experten einen teilstrukturierten Fragebogen zur schriftlichen Beantwortung. Der Interviewleitfaden beinhaltete ausschließlich offene Fragen. In einem zweiten Schritt wurden digitale Einzelinterviews^2^ von circa 30 bis 120 min durchgeführt. Dadurch konnten sowohl in der schriftlichen Befragung aufgetretene Fragen auf Seiten der Experten geklärt als auch Verständnis- bzw. Rückfragen in Bezug auf die schriftlichen Antworten diskutiert werden (Steffen und Doppler 2019). Die Antworten der Einzelinterviews sowie die Interviewunterlagen wurden von den jeweiligen Experten nach entsprechender Prüfung für die Analyse freigegeben.

Die Auswertung der Experteninterviews geschah anhand einer qualitativen Inhaltsanalyse, um das CX-Management der NÜRNBERGER Versicherung möglichst sinnrekonstruierend abzubilden und zu interpretieren (Döring und Bortz 2016; Gläser und Laudel 2010). Die Analyse erfolgte mittels Extraktion, d. h. den Interviews wurden relevante Informationen entnommen und diese anschließend getrennt von den ursprünglichen Antworten weiterverarbeitet (Gläser und Laudel 2010). Das Kategoriensystem für die Extraktion wurde zuerst auf Basis der beschriebenen Teilbereiche und Inhalte des CEM-Prozesses nach Mayer-Vorfelder (2012a) deduktiv erstellt und anschließend anhand der Expertenaussagen induktiv erweitert (Döring und Bortz 2016).

## Ergebnisse der Experteninterviews

Die folgenden Abschnitte beschreiben die aus der qualitativen Studie resultierenden Erkenntnisse. Zunächst wird die Wahrnehmung und Einordnung der Experten hinsichtlich des CEM in der Versicherungsbranche zusammengefasst. Anschließend erfolgt die Darstellung des CX-Managements der NÜRNBERGER Versicherung auf Basis des von Mayer-Vorfelder (2012a) vorgeschlagenen Managementprozesses für das CEM im Dienstleistungsbereich.

### Customer Experience Management in der Versicherungsbranche

#### Stellenwert des Customer Experience Managements

Insgesamt schreiben alle Experten dem CEM einen hohen bis sehr hohen betriebswirtschaftlichen Stellenwert für die Versicherungsbranche zu. Zurückgeführt wird die Relevanz dieses Managementansatzes auf zwei grundsätzliche Entwicklungen. Zum einen sind die Weiterentwicklungsmöglichkeiten der Versicherungsbranche im Hinblick auf die reine Produkt- und Leistungsebene bereits relativ ausgereizt bzw. begrenzt, weshalb eine Differenzierung über die Produkte nicht mehr nachhaltig möglich ist. Versicherungsprodukte sind austauschbar, leicht vergleichbar und schnell kopiert. Eine Differenzierung findet daher mehr denn je über die Serviceleistungen rund um den eigentlichen Versicherungsschutz statt.

Parallel steigen aufgrund der anhaltenden Digitalisierung und der sog. ‚Amazonisierung‘ die Erwartungen und Ansprüche der Kunden an die Versicherungsunternehmen. Die durch den Online Versandhändler Amazon und andere innovative Unternehmen neu gesetzten Standards im Hinblick auf Bearbeitungsgeschwindigkeit, Same- und Next Day Delivery sowie Tracking von Bestellungen, erwarten die Kunden nun auch in anderen Branchen. Dies ist auch in der Versicherungsbranche zu beobachten. Die Digitalisierung ermöglicht es den Kunden zudem, mit mobilen Endgeräten rund um die Uhr und ortsunabhängig auf alle Informationen zuzugreifen. Gleichzeitig wird der Alltag schnelllebiger und es bleibt weniger Zeit für Themen wie Versicherungen. Ein Beispiel für diese Entwicklungen stellt die Erwartungshaltung der Kunden an die Bearbeitungsdauer ihrer Anliegen über verschiedene Kommunikationsmedien hinweg dar. Kunden erwarten z. B. die Beantwortung einer E‑Mail innerhalb von 48 h, eines Briefs innerhalb von einer Woche und eines Anrufs innerhalb von drei bis fünf Minuten. Zukünftig könnten auch Themen wie Rund-um-die-Uhr-Erreichbarkeit und Echtzeit-Antworten auf verschiedenen Kanälen noch relevanter im Entscheidungsprozess für einen Versicherer und seine Produkte werden. Zugleich steigen die Erwartungen der Kunden an Self-Services: *„Schnell mal eine Versicherung abschließen, schnell mal einen Haftpflichtschaden melden oder nur über eine Chatfunktion am Handy die Schadenbilder hochladen und reguliert bekommen. Das ist die digitale Erwartungshaltung der Kunden“ (Exp_07).*

Diese Entwicklungen werden von der COVID-19-Pandemie seit Anfang 2020 verstärkt und beschleunigt. Gleichzeitig ist festzustellen, dass *„der Wunsch nach persönlichen Kontakten immens steigt. […] Wir beobachten die Entwicklung […] seit Anfang des Jahres [2020] und wir gehen davon aus, dass es sich im Kommunikationsverhalten auf breiter Ebene manifestieren wird“ (Exp_02)*. Diese Beobachtung bestätigt eine aktuelle Studie von Sirius Campus (2020) zu „Multi-Channel-Management und Kontaktpräferenzen in Corona-Zeiten“ in Bezug auf die gesamte Versicherungsbranche. Auch diese Entwicklung stellt eine Erkenntnis für die Ausgestaltung der Kundenkontakte im CEM dar, da wieder häufiger persönliche Kontakte mit den Vertriebspartnern stattfinden müssen.

Es ist also heutzutage umso wichtiger, sich mit hochdifferenzierten Kundenerwartungen auseinanderzusetzen und sich danach auszurichten, um den Ansprüchen und Bedürfnissen der Kunden langfristig gerecht zu werden. Eine vielversprechende Möglichkeit zur Bewältigung dieser Herausforderung sehen viele Versicherungsunternehmen in dem CEM.

#### Status quo des Customer Experience Managements

Seit 2015 ist die Anzahl der Versicherungsunternehmen, die bereits erfolgreich ein CEM implementiert haben oder derzeit aufbauen, stark gestiegen. Die stetig wachsende Relevanz wird unter anderem dadurch deutlich, dass das CEM mittlerweile auf Top-Management Ebene als Fokusthema priorisiert vorangetrieben und die Kundenzentrierung vorgelebt wird.

Der schnelle und erfolgreiche Aufbau eines CEM ist dabei sowohl von der systemtechnischen Infrastruktur und den verfügbaren Ressourcen als auch von der Bereitschaft des einzelnen Unternehmens für grundlegende Veränderungen abhängig. Vor allem der letztgenannte Punkt trägt entscheidend zum Erfolg dieses Managementansatzes bei. Insgesamt beobachten *Exp_03* und *Exp_04*, dass die Versicherungsunternehmen im Rahmen des Aufbaus eines CEM, abhängig von den jeweiligen Zielen, die notwendigen Veränderungen im Unternehmen in unterschiedlichem Umfang und mit verschiedenen Herangehensweisen vorantreiben. Häufig wird in den Unternehmen erst langsam an den Strukturen gearbeitet sowie Funktionen und Aufgabenbeschreibungen verändert bzw. neu geschaffen. Eine weitere wichtige Veränderung, der es im Zuge des Aufbaus eines CEM bedarf, ist der Kulturwandel. Dieser sollte ein Verständnis für die engen Verbindungen zwischen der Kundenzufriedenheit, dem CEM und der Kundenorientierung schaffen: *„Die Zufriedenheit wird erzielt, wenn Kundenorientierung herrscht. Und um kundenorientiert handeln zu können, muss sich der Versicherer damit befassen, was Kunden von ihm erwarten“ (Exp_09)*. Einen Schwerpunkt des Kulturwandels stellt deshalb die Mobilisierung der Mitarbeiter (siehe Abschn. 4.2.2) dar: *„Hier wird durch Kommunikationskampagnen, Kundenbüros, Aktionstage, Kundentage, ‚Stimme des Kunden‘ und ähnliches mehr der Kunde in den Alltag der Mitarbeiter geholt“ (Exp_04)*.

Die Studie KUBUS Privatkunden^3^ 2021 der MSR Consulting Group GmbH zeigt, *„dass die Bemühungen der Häuser in Bezug auf das Customer Experience Management [weiter] Früchte tragen. Unternehmen, die aktiv daran und damit arbeiten, verbessern sich. Damit verbessert sich auch der Markt insgesamt“ (Exp_03).* Die Notwendigkeit resultiert laut *Exp_03* unter anderem aus Mitbewerbern, die durch das Erreichen solch hoher Werte der Kundenzufriedenheit und der Weiterempfehlungsbereitschaft (Net Promoter Scores nahe dem Niveau von Apple & Co.) zeigen, dass viel Potenzial für Verbesserungen durch den Einsatz von und die Arbeit mit CEM vorhanden ist. Der Handlungsdruck hinsichtlich des CEM resultiert daher aus dem Markt, der die Geschwindigkeit für Veränderungen vorgibt.

Zusammenfassend lässt sich mit Blick auf die genannten kunden- und markseitigen Entwicklungen im Versicherungsmarkt festhalten: *„Die Herausforderung besteht darin, dass man als Unternehmen sich immer wieder auf wechselnde Szenarien einstellen muss, wenn beispielsweise neue Technologien oder ein Wandel Unruhe in das Unternehmen bringen“ (Exp_04)*. Wie Versicherungsunternehmen sich den aktuellen Herausforderungen stellen, wird im Folgenden exemplarisch anhand der NÜRNBERGER Versicherung und deren Aufbau, Implementierung sowie Ausgestaltung ihres CX-Managements näher beleuchtet. An dieser Stelle ist darauf hinzuweisen, dass der Fokus dieser Studie auf den Bemühungen der Zentrale im Hinblick auf die Steigerung der Kundenzufriedenheit und Kundenzentrierung durch das CX-Management liegt. Der Vertrieb, der für den Service-Versicherer schon immer einen bedeutenden Touchpoint darstellt und eine entscheidende Rolle für die Kundenzufriedenheit spielt, wird daher nicht gesondert betrachtet und zählt im Folgenden zu den an der Customer Experience sowie Customer Journey des Kunden beteiligten Bereichen des Unternehmens.

### CX-Management bei der NÜRNBERGER Versicherung

Der Aufbau des CX-Managements der NÜRNBERGER Versicherung wurde 2016 im Rahmen der Markenneupositionierung gestartet. Im Zuge des Relaunches durchgeführte Kundenbefragungen ergaben, dass die Markenversprechen für die Kunden oft nicht erlebbar waren. Dies führte in Folge zu niedrigen Kundenzufriedenheitswerten in der KUBUS Studie Privatkunden und dem Vorstandsbeschluss, die Zufriedenheit der NÜRNBERGER Kunden messbar zu verbessern. Die engen Verbindungen zwischen dem CEM, der Kundenzufriedenheit und der Marke, die von Anfang an das CX-Management geprägt haben, werden von *Exp_02* wie folgt beschrieben: *„Customer Experience Management ist eine Maßnahme zur Steigerung der Kundenzufriedenheit und diese Kundenzufriedenheit ist wiederum unerlässlich, um einen starken Markenwert zu erzielen“*. Die neuen Markenwerte ‚klar, unkompliziert, solide, verlässlich, unabhängig und menschenzentriert‘ spiegeln eine klare Kundenzentrierung des Unternehmens wider.^4^ Seit 2018 ist das CX-Management als eigene Organisationseinheit auf operativer Ebene fest in der NÜRNBERGER Versicherung installiert. Nach erfolgreicher Implementierung in den Sparten Kfz-Versicherung, Sach‑, Haftpflicht- & Unfallversicherung, Berufsunfähigkeitsversicherung und Leben- & Rentenversicherung erfolgte im Jahr 2021 der Aufbau des CX-Managements im Geschäftsbereich Krankenversicherung.

Heute hat das CX-Management bereits einen sehr hohen Stellenwert sowie eine hohe Akzeptanz in der NÜRNBERGER Versicherung. Der Stellenwert wird beispielweise durch die Schaffung einer Organisationseinheit mit entsprechend ausgebildeten Mitarbeitern (siehe Abschn. 4.2.2) ersichtlich, die den notwendigen Austausch aller Schnittstellen, die an Kundenprozessen mitwirken, fördern und als Ansprechpartner für alle Mitarbeiter des Unternehmens greifbar sind. Zudem hat sich das CX-Management zu einem *„Hebel entwickelt, der verstaubte Kundenprozesse in Bewegung setzt“ (Exp_09)*. *Exp_08* ist der Überzeugung, dass neue Anforderungen und Verbesserungsmaßnahmen, die über das CX-Management eingebracht werden, im Unternehmen und in den Abteilungen einen höheren Stellenwert haben, stärker priorisiert sowie schneller umgesetzt werden. Die Grundlage hierfür liefern die von der zentralen NÜRNBERGER Marktforschung laufend entlang der Customer Journey zu allen wichtigen Kontaktpunkten erhobenen und analysierten *„Ergebnisse der [Kunden-]Umfragen und die Zufriedenheitswerte. Über die kann man […] besser argumentieren und die Dringlichkeit von Maßnahmen darstellen, beispielsweise über deutlich sichtbare Divergenzen“ (Exp_08)*.

**Figure 1:**
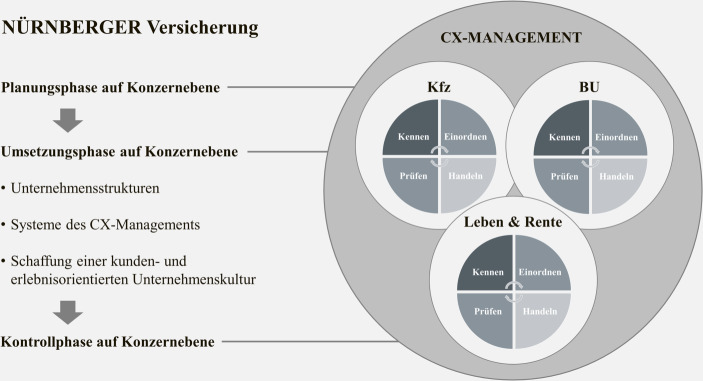

Wie das CX-Management aufgebaut ist und wie die Arbeit dieses Managementansatzes abläuft wird im Folgenden ausführlich beleuchtet. Die nachfolgenden Abschnitte sind dabei in Anlehnung an den von Mayer-Vorfelder (2012a) vorgeschlagenen Managementprozess für das CEM im Dienstleistungsbereich strukturiert. Die Experteninterviews haben gezeigt, dass der Managementprozess nach Mayer-Vorfelder (2012a) auf Konzernebene sowie je Geschäftsbereich im Rahmen des Closed Loops der CX-Methodik abläuft. Abb. 1 veranschaulicht die Erkenntnis dieser beiden parallel ablaufenden Managementprozesse innerhalb der NÜRNBERGER Versicherung. Eine Analysephase auf Konzernebene entfällt aufgrund der *„regulatorisch vorgeschriebene[n] Spartentrennung, die wiederum die gesamtheitliche Kundensicht erschwert“ (Exp_04)*, während die Analysephase im Zuge des Aufbaus des CX-Managements für einen Geschäftsbereich durch die Entwicklung und Anpassung der jeweiligen Kundenreisen annähernd nach Mayer-Vorfelder (2012a) abläuft. Die Planungs- und Umsetzungsphase treffen wiederum auf Konzernebene auf Mayer-Vorfelder (2012a) zu, finden aber auch im Closed Loop in der Planung und Umsetzung von Optimierungsmaßnahmen Anwendung. Der Closed Loop insgesamt kann ebenfalls der Umsetzungsphase nach Mayer-Vorfelder (2012a) zugeordnet werden. Schlussendlich findet die Kontrollphase des CX-Managements auf Konzernebene im Hinblick auf die Gesamtzufriedenheit mit der NÜRNBERGER Versicherung im Marktvergleich und mit Blick auf weitere Erfolgsgrößen entlang der Customer Journey der Kunden in spartenindividueller Ausprägung statt. Innerhalb des Closed Loops je Geschäftsbereich werden durch die Kundenbefragungen die Optimierungsmaßnahmen regelmäßig kontrolliert.

Nachfolgend werden die Phasen des CX-Managements auf Konzernebene vorgestellt, wobei der Closed Loop des CX-Managements in Abschn. 4.2.2 Anwendung findet.

#### Ziele und Planung des CX-Managements

Das CX-Management der NÜRNBERGER Versicherung wird bereits seit 2016 als zentraler Teil der Erfolgsfaktoren des Unternehmens angesehen und verfolgt als Strategie-Baustein das übergeordnete Konzernziel und Fokusthema der ‚ausgezeichneten Kunden- und Vermittlerzufriedenheit‘. Vor dem Hintergrund aller Maßnahmen und Instrumente zur Verbesserung der Kundenzufriedenheit (z. B. Schulungen, Digitalisierung von Kundenprozessen), die im Unternehmen umgesetzt werden, ist der Aufbau und die Linienarbeit mit dem CX-Management das umfangreichste und strategisch wichtigste Programm zur Erreichung des Konzernziels. Dem CX-Management wird zudem in der täglichen Arbeit der höchste und messbarste Einfluss auf die Kundenzufriedenheit zugeschrieben. Die Kundenzufriedenheit stellt somit die Basis sämtlichen Handelns im Rahmen des CX-Managements dar.

Mit Blick auf das Konzernziel der Kundenzufriedenheit verfolgt das CX-Management das konkrete Ziel der Verbesserung der Kundenerlebnisse in allen Bereichen, die eine Schnittstelle zum Kunden darstellen, welches auch in der Literatur beschrieben wird (u. a. Bruhn und Hadwich 2012; Lemon und Verhoef 2016; Meyer und Schwager 2007). Als Grundlage hierfür dient dem CX-Management das Kundenfeedback aus Befragungen, denn *„ohne die Meinung unserer Kunden zu kennen, wissen wir schließlich nicht, wo unsere Handlungsfelder liegen“ (Exp_05)*. Daran knüpfen auch die von *Exp_03* genannten wesentlichen Ziele des CEM an, die sich ebenfalls bei der NÜRNBERGER Versicherung sowie in der Umsetzungsphase nach Mayer-Vorfelder (2012a) wiederfinden:

„**Insights:** Perfektes Kundenverständnis über das gesamte Unternehmen hinweg

**Action:** Aufstellung und Mobilisierung der Organisation, so dass diese Kundenbedürfnisse optimal erfüllt werden können; Umsetzung der Insights in einem kontinuierlichen Verbesserungsprozess

**Agility:** Erzeugung einer kundenzentrierten Haltung, die für den Kunden in der Interaktion spürbar ist und die Bereitschaft für eine permanente Anpassung der Organisation, ausgerichtet auf die Kundenanforderungen, schafft“.

Nach der Zielfestlegung empfiehlt Mayer-Vorfelder (2012a) im Zuge der strategischen Erlebnisplanung die Festlegung eines übergeordneten Erlebnismotivs. Ein eigens für das CX-Management geschaffenes Erlebnismotiv gibt es bei der NÜRNBERGER Versicherung nicht, jedoch wird sich stark an der Markenvision ‚Wir wollen, dass Menschen einfach den passenden Schutz finden‘ orientiert, denn *„jedes Erlebnis, dass der Kunde hat, ist ein Erlebnis mit der Marke. Von daher sollten die [Marke und das Customer Experience Management] zusammengebracht werden. Bei der NÜRNBERGER ist das Besondere, dass das Customer Experience Management aus dem Markenrelaunch gestartet ist und man diese Verbindungen zwischen Marke und Kundenerlebnis von Vornherein im Blick hatte“ (Exp_03)*. Das CX-Management setzt also mit dessen Arbeit an der Erfüllung der Markenvision sowie des Markenversprechens an und hat damit einen wesentlichen Anteil an der Steigerung des Markenwertes. Dieser besteht wiederum aus der eigentlichen Markenwahrnehmung und zu einem weitaus größeren und wichtigeren Teil aus dem Markenerlebnis, welches ein Versicherungsunternehmen bei allen Kundenkontakten liefert. Auf operativer Planungsebene arbeitet die NÜRNBERGER Versicherung hinsichtlich des Customer Experience Designs im Vergleich zu dem von Mayer-Vorfelder (2012a) vorgeschlagenen Einsatz des Service Experience Blueprints ausschließlich mit Customer Journeys. Diese werden in der Umsetzungsphase des CX-Managements entwickelt.

#### Umsetzung des CX-Managements bei der NÜRNBERGER Versicherung

Die durch Mayer-Vorfelder (2012a) vorgenommene Dreiteilung der Umsetzungsphase des CEM in Strukturen, Systeme und Kultur lässt sich auch für das CX-Management der NÜRNBERGER Versicherung auf Konzernebene abbilden. Außerdem stellt der Closed Loop des CX-Managements, wie bereits in Abschn. 4.2 erläutert, nochmals einen Managementprozess innerhalb der Umsetzungsphase auf Ebene der Geschäftsbereiche dar, worauf ebenfalls in diesem Abschnitt eingegangen wird.

##### Unternehmensstrukturen

*Abteilung CX-Management & CX-Aufbauorganisation*

Das CX-Management als eigene Organisationseinheit wurde 2018 in der Aufbauphase im Bereich Marketing geschaffen und besteht derzeit aus sechs ausgebildeten CX-Managern und einem Leiter CX-Management. Die Organisationseinheit arbeitet konzernübergreifend mit allen Bereichen, die Touchpoints mit den Kunden zu verantworten haben, zusammen. Da ein so grundlegendes Vorhaben wie ein CEM nicht von den CX-Managern alleine vorangetrieben werden kann, hat das Unternehmen in all diesen Bereichen rund 130 Kundenlotsen und Touchpointverantwortliche benannt, methodisch ausgebildet und dadurch mit einer CX-Aufbauorganisation sehr schnell die notwendigen Strukturen für ein erfolgreiches CEM geschaffen.

Die CX-Manager betreuen jeweils als Zweierteam eine bzw. mehrere Sparten im Privatkundenbereich. Der vielfältige Aufgabenbereich der CX-Manager, die als bereichsübergreifende Ansprechpartner für das CX-Management und damit als dessen Multiplikatoren innerhalb des Unternehmens angesehen sind, besteht aus drei Kernelementen. Zu den Linienaufgaben zählt einerseits *„gemeinsam mit [den] Kundenlotsen und Touchpointverantwortlichen Optimierungsmaßnahmen anhand [der] Kundenfeedbacks abzuleiten, zu entwickeln, zu steuern und in der Umsetzung der Maßnahmen zu unterstützen“ (Exp_05)*. Die zweite Kernaufgabe umfasst u. a. die Sicherstellung einer konsequenten, konzernweit einheitlichen Anwendung sowie der Weiterentwicklung der CX-Methodik, die tägliche Arbeit mit (u. a. Analyse von Kundenfeedback sowie Ableitung von Handlungsfeldern) und die Pflege des CX-Mess-Systems (z. B. Optimierung Customer Journeys, Qualitätssicherung) als auch die Steuerung der regelmäßigen Kundenreiseworkshops. Neben den Linientätigkeiten arbeiten die CX-Manager drittens in diversen Konzernprojekten und ad-hoc-Maßnahmen mit, in denen sie die Kundensicht einbringen und vertreten.

Für die Jobpositionen des CEM gibt es mannigfaltige und von Unternehmen zu Unternehmen variierende Begriffe. Der Begriff Kundenlotse wurde während der Implementierung des CX-Managements von der NÜRNBERGER Versicherung gemeinsam mit MSR Consulting aufgrund dessen passender Bildsprache entwickelt. Andere Unternehmen nennen Mitarbeiter dieser Position beispielsweise Kundennavigatoren oder Markenbotschafter. Die Kundenlotsen der NÜRNBERGER Versicherung üben mit einem Aufwand von ca. 20 % der Arbeitszeit die Position als Botschafter der Kundensicht und des CX-Managements in ihrer jeweiligen Abteilung aus. Zu den Aufgabenfeldern der Kundenlotsen zählen u. a. die Koordination sowie Unterstützung bei der Entwicklung und Umsetzung von Verbesserungsmaßnahmen. Zudem sind sie für die Vorbereitung und Durchführung der monatlichen Regelworkshops mit den Touchpointverantwortlichen der jeweiligen Organisationseinheit und den CX-Managern zuständig. Die Touchpointverantwortlichen sind im Rahmen ihrer Funktion, die sie als Zusatzfunktion zu ihrer Linienarbeit freiwillig ausüben, zuständig für das Kundenerlebnis am jeweiligen Touchpoint, die Rückmeldung von Optimierungsansätzen und die Unterstützung bei der Maßnahmenentwicklung.

Neben dem Dreiklang aus CX-Managern, Kundenlotsen und Touchpointverantwortlichen spielt das Team Market Research rund um die Projektleitung für die Entwicklung und Implementierung des CX-Managements eine wichtige Rolle. Dieses verantwortet den Aufbau des konzernübergreifenden CX-Mess-Systems, die Durchführung der laufenden Kundenzufriedenheitsbefragungen sowie die Datenverarbeitung und das Reporting. Market Research liefert aus den Ergebnissen Arbeitshilfen wie Treiberanalysen und regelmäßige CX-Reports. Außerdem entwickelt es gemeinsam mit Projektteams aus allen Geschäftsbereichen das CX-Management für neue Geschäftsfelder einschließlich der Erfolgsmessung der Verbesserungsmaßnahmen in Bezug auf die Kundenzufriedenheit.

*Personalmanagement*

Alle Mitarbeiter der CX-Aufbauorganisation haben eine mehrtägige Ausbildung durchlaufen, um deren Verständnis für Kundenzufriedenheit und -zentrierung zu stärken sowie in verschiedenen Workshops die Arbeit mit der CX-Methodik zu erlernen. *„Am Ende der Ausbildung sollen die Mitarbeiter dann ein klares Bild haben von ‚Was muss ich tun? Wofür sind wir da und wo ist der Nutzen unseres Handelns für den Kunden? Wie bin ich ein USP?‘. Meist funktioniert das am Ende der Ausbildung viel besser als am ersten Tag. Und das zeigt uns, dass die Mitarbeiter viel mitgenommen haben und sich ihrer Aufgabe im Customer Experience Management durch die Ausbildung viel mehr bewusstwurden“ (Exp_04)*. Die Ausbildung stellt eine Zusatzausbildung dar, die mit einem Zertifikat belohnt wird. Den Positionen wird dadurch eine Wertigkeit und Anerkennung entgegengebracht, die auch die Motivation zur Teilnahme bei anderen Mitarbeitern wecken kann. Die Vorstellung des CX-Managements ist mittlerweile zudem ein Teil des Onboardings neuer Mitarbeiter, um die Mobilisierung im Sinne der Kundenzentrierung vom ersten Tag an voranzutreiben.

*Verankerung auf der Vorstandsebene*

Zum 01. Januar 2021 wurde das CX-Management in das neu geschaffene Vorstandsressort ‚Kundenbeziehungsmanagement und Operations‘ umgesiedelt. Durch den Aufbau des neuen Vorstandsressorts und der dortigen Ansiedlung der Organisationseinheit als Treiberfunktion im Bereich ‚Kundenbeziehungsmanagement‘ erhält das CX-Management, aber auch die Kundenzentrierung, nach Meinung der Experten einen neuen und präsenteren Stellenwert im Unternehmen.

##### Systeme des CX-Managements

Die NÜRNBERGER Versicherung verwendet im Rahmen des CX-Managements zwei analytische Systeme: ein Customer Feedback System, über das die Kundenbefragungen zur Analyse der Kundenzufriedenheit realisiert werden, sowie ein CX-Mess-System, in dem unter anderem die Kundenbewertungen tagesaktuell in einem eigens entwickelten Dashboardsystem abgebildet werden. Hinsichtlich des CX-Mess-Systems arbeitet die NÜRNBERGER Versicherung aktuell mit einem digitalen Umfragetool. Die Ergebnis-Reports werden der CX-Aufbauorganisation auf einer cloudbasierten CX-Plattform zur Verfügung gestellt, die für ein ganzheitliches Closed-Loop-CX-Management an die Bedürfnisse und Vorstellungen des Unternehmens angepasst wurde. Die CX-Plattform stellt unter anderem Funktionen für Customer Journey Mapping, Touchpoint Management und Kundenbeziehungsmanagement zur Verfügung und wird seit dessen Implementierung als tägliches Tool des CX-Managements in der sog. CX-Methodik eingesetzt. Für den Aufbau und den Betrieb der Systeme ist Market Research verantwortlich.

*CX-Methodik der NÜRNBERGER Versicherung*

Der Aufbau und die Linienarbeit des CX-Managements erfolgen bei der NÜRNBERGER Versicherung einzeln je Geschäftsbereich und anhand der sogenannten CX-Methodik. Eine wichtige Aufgabe bei der Neuentwicklung des CX-Managements für einen Geschäftsbereich ist die Entwicklung der Personas und ihrer Customer Journeys. Personas sind Prototypen von Zielgruppen bzw. (marktforschungs-)datenbasierte Darstellungen der für ein Unternehmen typischen Kunden(‑segmente) und deren spezifischer Eigenschaften und Bedürfnisse (Lemon und Verhoef 2016; Teixeira et al. 2012). Für jede Persona wird eine ‚typische‘ Customer Journey entwickelt. Dies geschieht unter Anwendung des Customer Journey Mappings mit allen an der Customer Journey beteiligten Bereichen und mit dem Ziel, alle potenziellen (in)direkten Touchpoints eines Kunden mit dem Unternehmen über die Vorkauf‑, Kauf- und Nachkaufphase hinweg für den entsprechenden Geschäftsbereich grafisch abzubilden (Lemon und Verhoef 2016; Rosenbaum et al. 2017). Die NÜRNBERGER Versicherung arbeitet mit sechs Personas, die sich marktüberdurchschnittlich für das Unternehmen interessieren oder dessen Produkte präferieren. Für das kundenorientierte CX-Management ist dieses Wissen besonders wertvoll, da so möglichst exakt auf die Bedürfnisse, Erwartungen und Wünsche der Personas eingegangen werden kann, beispielsweise mit Blick auf den Familienstand, den Absicherungsbedarf und die Kontakterwartungen sowie -frequenz. Die Customer Journeys werden daher für jede Persona erstellt, digitalisiert und in dem CX-Mess-System abgebildet. Sie sind in vier Reiseabschnitte gegliedert. Je Reiseabschnitt werden in dem CX-Mess-System die Teiljourney-Phasen (z. B. Wahrnehmung), die einzelnen Kundenaktivitäten (z. B. ‚Ich recherchiere‘) sowie die jeweils zugehörigen, einzelnen Touchpoints mit der NÜRNBERGER Versicherung (z. B. ‚Erstes Beratungsgespräch persönlich‘) übersichtlich dargestellt. Die farbliche Kennzeichnung der Touchpoints definiert eindeutige Verantwortlichkeiten interner Abteilungen bei der Entwicklung von Optimierungsmaßnahmen.

Die Touchpoints werden im Zuge des Customer Journey Mappings je Sparte, je Kundenreiseabschnitt sowie je Persona festgehalten und in dem CX-Mess-System abgebildet. Welche die aus Kundensicht wichtigsten und relevantesten Touchpoints für die Gesamtzufriedenheit sind, wird mit Hilfe von Treiberanalysen von Market Research jährlich neu berechnet. Treiber- bzw. Wirkungsmodelle werden im Marketing eingesetzt, um zu analysieren, wie sich hypothetische Änderungen in der Bewertung einzelner Teilkomponenten des Modells auf das nächste Glied innerhalb einer Erfolgskette, z. B. die Gesamtzufriedenheit, auswirken (Bruhn und Hadwich 2021; Büschken et al. 2013) und welche Relevanz ihnen somit zugeschrieben werden sollte. Die Basis für diese aufwändigen Regressionsanalysen, die in der gesamten Versicherungswirtschaft von den Unternehmen mit einem CEM betrieben werden, bilden die Ergebnisse der Kundenbefragungen. Die Kategorisierung der Touchpoints durch die Treiberanalysen wird bei der NÜRNBERGER Versicherung als elementare Grundlage des betriebswirtschaftlichen Handelns eingestuft, da sie u. a. einen starken Einfluss auf die Entscheidungen hinsichtlich der Priorisierung von Projekten des CX-Managements haben.

Auch wenn der aktuelle Fokus der Kundenerwartungen bei den Themen Multi-Channel-Erreichbarkeit, Service und Verständlichkeit der Unterlagen verortet werden kann, so erwarten die Experten der NÜRNBERGER Versicherung eine stetige Zunahme der Anzahl und Wichtigkeit digitaler Touchpoints, wie beispielsweise dem Online-Kundenportal und Self-Services. Das Unternehmen hat daher, verstärkt durch die COVID-19-Pandemie, im letzten Jahr einige Touchpoints digitalisiert (z. B. digitale Antragsunterschrift). Gleichzeitig werden aber ,alte‘ Touchpoints, wie der persönliche Kontakt zum Vermittler, mittelfristig nicht verschwinden. Ganz im Gegenteil. Die bereits erwähnte Studie von Sirius Campus (2020) ergab, dass Versicherungskunden vor allem hinsichtlich Beratung und Abschluss wieder mehr persönliche Kontakte wollen, während Unterstützungsangebote und Services auch digital stattfinden können. Im Allgemeinen nimmt zudem der Kontaktwunsch zur Versicherung selbst bzw. der Zentrale ab (Sirius Campus 2020). Interesse besteht immer mehr an persönlichem Kontakt zum Versicherungsvermittler (Sirius Campus 2020), d. h. Personen, die man kennt anstatt ‚anonymisierte‘ Kontakte mit der Zentrale.

Die Relevanz der Touchpoints lässt sich jedoch nicht über alle Versicherungsunternehmen hinweg verallgemeinern und variiert beispielsweise ganz grundsätzlich zwischen einem Direktversicherer ohne eigene Vertriebspartner und einem Service-Versicherer. Dahingegen wird den Touchpoints des sog. Moment of Truth in der gesamten Versicherungswirtschaft eine hohe Relevanz zugesprochen. Im Allgemeinen bezeichnet der Moment of Truth dabei bestimmte Schlüsselmomente des Kontakts zwischen einem Kunden und einer Marke (Moran et al. 2014), die über den Erfolg oder das Scheitern des Unternehmens entscheiden können (Carlzon 1987). *Exp_02 *sieht in dem Moment of Truth zwei wesentliche Einflussgrößen auf die Kundenzufriedenheit: *„Die Digitalisierung, gerade bei der Schadenregulierung, spielt da eine ganz ganz große und wichtige Rolle. Weil [dort] geht es um Geschwindigkeit. Es ist auf der einen Seite die Digitalisierung, heißt die [sofortige] Bereitstellung von Informationen, die Datenverarbeitung [z.* *B. elektronische Rechnungsprüfung] etc. Auf der anderen Seite ist der Moment of Truth aber auch stark von dem menschlichen Bedürfnis nach Hilfe und Betreuung geprägt. Wir machen beides“*. Trotz der Wichtigkeit des Moment of Truth, erleben pro Jahr, nach Auswertung der NÜRNBERGER Versicherung, nur etwa 10 % der Kunden einen Schaden- oder Leistungsfall, während der erste Kundenreiseabschnitt bis zum Vertragsabschluss wiederum von jedem Versicherungskunden durchlebt wird. Die Customer Experience bis zum Vertragsabschluss ist deshalb prägend für das Image eines Versicherers und die gesamte Kundenbeziehung, weshalb es aus Sicht von *Exp_02 *ratsam ist *„bei der Planung vor allen Dingen auf die Zufriedenheit der Kunden mit den einzelnen Kontaktpunkten bis […] sie ihren Versicherungsvertrag in den Händen halten den Schwerpunkt zu legen“*.

**Figure 2:**
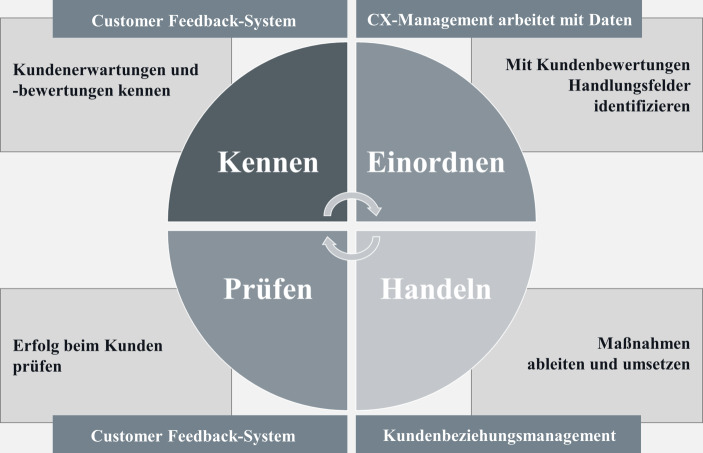

Die CX-Methodik für die Linienarbeit sieht vor, in einem Closed Loop alle Techniken und Methoden des CX-Managements zu vereinen. Die einzelnen Abschnitte des Closed Loops (siehe Abb. 2) sollen im Folgenden näher erläutert werden. Nachdem die Customer Journeys für eine Sparte entwickelt wurden, werden passgenaue Kundenbefragungen entwickelt und laufend durchgeführt. Die Kundenbewertungen sind dabei die Grundlage der Arbeit des gesamten CX-Managements und die Basis aller Maßnahmen zur Optimierung der Customer Experience: *„Denn meist sagt uns der Kunde ganz genau, wo unser Service nicht funktioniert und an welcher Stelle er unzufrieden ist. Mit diesen Informationen können Maßnahmen definiert werden, die den Service stetig verbessern“ (Exp_07)*. Mit Blick auf die Schaffung einer kunden- und erlebnisorientierten Unternehmenskultur dient das Kundenfeedback des Weiteren als eine Art Währung, d. h. die Erwartungen und Bedürfnisse der Kunden werden dadurch sowohl für Vorstandsmitglieder als auch Mitarbeiter aller Ebenen erlebbar und greifbar. Das Feedback der Kunden wird über das Customer Feedback System in Form von quantitativen anlassbezogenen Befragungen standardisiert und vollautomatisiert eingeholt und in das CX-Mess-System eingespeist. Dies geschieht jeweils nach den ersten drei Kundenreiseabschnitten. In einem zeitlichen Abstand von wenigen Tagen nach dem Kontakt, zur Gewährleistung einer guten Erinnerungsfähigkeit des Kunden, erhalten so beispielsweise Neukunden nach dem Vertragsabschluss, Bestandskunden u. a. nach einer inhaltlichen Vertragsänderung oder einem Kundenservice während der Vertragslaufzeit und nach der Schadenregulierung eine Einladung zur Teilnahme an der Befragung. Die Kundenbefragungen erfolgen mehrheitlich schriftlich (Response bei ca. 10–27 %). Die Möglichkeit der Online-Teilnahme wird nur von einem kleinen Teil der Kunden genutzt. Weiterhin setzt das NÜRNBERGER Communication Center nach einem Telefonkontakt eine automatische Abfrage zur Einholung der Kundenfeedbacks hinsichtlich Erreichbarkeit und Zufriedenheit mit der telefonischen Erledigung von Anliegen ein, deren Ergebnisse ebenfalls in das CX-Mess-System übertragen werden. Ganz im Sinne der Kundenzentrierung geht die NÜRNBERGER Versicherung noch einen Schritt weiter und bindet die Kunden aktiv in die Gestaltung der zukünftigen Customer Experience mit ein: *„Wir wollen da noch mehr wissen. Parallel zu dem CX-Mess-System unterhält die NÜRNBERGER eigene Kunden-Communities […], mit denen wir dann […] in den Austausch und die Diskussion gehen, in die tiefere Befragung. Wir machen [zum Beispiel] Produkttests über die Kunden-Communities. Wir gehen soweit runter, dass wir sogar Prospekte vom Kunden bewerten und beurteilen lassen. Die Kunden sind da bei uns in den [Entwicklungsprozess] sehr stark involviert“ (Exp_02)*. Diese aktive und tiefergehende Einbindung und Befragung der Kunden zur Anreicherung der quantitativen Kundenfeedbacks beschreiben Homburg et al. (2017) in Bezug auf das CEM als eine von vier relevanten Fähigkeiten eines Unternehmens (siehe Abschn. 2.2), um die Customer Experience durch Anpassung der Touchpoints kontinuierlich zu verbessern bzw. zu erneuern.

Die Arbeit mit den Ergebnissen der Kundenbefragungen (‚Einordnen‘; siehe Abb. 2) und die Ableitung der entsprechenden Maßnahmen zur Verbesserung der Customer Experience (‚Handeln‘; siehe Abb. 2) erfolgen sowohl in der kontinuierlichen Arbeit mit dem CX-Mess-System als auch in verschiedenen Workshops sowie anhand der CX-Reports. Die erhobenen Kundenfeedbacks sowie die entsprechenden Benchmarkdaten werden dafür in dem CX-Mess-System zusammengeführt und von den CX-Managern analysiert. Die Zufriedenheitswerte aus den Kundenfeedbacks sind dabei tagesaktuell. Zugriff auf das CX-Mess-System und damit einen Einblick in die Entwicklung der Kundenzufriedenheit haben die CX-Manager, die Kundenlotsen sowie zusätzlich Führungskräfte und Vorstände. In den einzelnen Dashboards und Dashlets wird ersichtlich, wie sich die Kundenzufriedenheit im aktuellen Jahr, in den zurückliegenden Jahren bzw. Monaten und im Wettbewerbsvergleich im Jahresdurchschnitt entwickelt hat. So kann auch der Erfolg von Optimierungsmaßnahmen nachträglich kontrolliert werden. Mit dem CX-Mess-System hat die NÜRNBERGER Versicherung somit ein Kennzahlensystem eingeführt, das es vor Implementierung des CX-Managements nicht gab. Obwohl bereits Kundenbefragungen durchgeführt wurden, werden die Daten erst mit Aufbau des CX-Managements derart effektiv und detailliert bereitgestellt. Die strategischen Kennzahlen leiten sich dabei aus den Konzernzielen ab, während die operativen Kennzahlen die Zufriedenheitswerte auf Ebene der einzelnen Touchpoints bzw. Kundenreiseabschnitte der Customer Journeys darstellen. Die Soll-Werte bzw. Zielwerte werden aufgrund des Konzernziels den Benchmarkdaten entnommen. Der Abgleich mit den Ist-Werten der Kundenzufriedenheit und somit die Zielerreichung werden in dem CX-Mess-System anhand von Tachos farblich angezeigt. So wird anhand der Ampelfarben auf einen Blick ersichtlich, bei welchen Touchpoints die NÜRNBERGER Versicherung über/auf Marktniveau (= grün), etwas unter dem Markt (= gelb) oder deutlich unter dem Marktdurchschnitt (= rot) liegt. Aus den beiden letzteren Ergebnissen werden schließlich Handlungsfelder abgeleitet: *„Eine Bewertung unter dem Marktdurchschnitt stellt für uns eine Divergenz dar, an der priorisiert gearbeitet werden muss. Bei einer Bewertung, die knapp unter dem Marktdurchschnitt liegt, sprechen wir von einer Optimierung – heißt: Diese Touchpoints werden v.* *a. beobachtet und bei Bedarf und je nach Kapazität weiterentwickelt. […] Die priorisierten Touchpoints und daraus abgeleiteten Maßnahmen sollten jederzeit auf unser übergeordnetes Ziel einzahlen“ (Exp_05)*. Daher werden die Divergenzen mit dem größten Einfluss auf die Kundenzufriedenheit, errechnet durch die Treiberanalysen, priorisiert angegangen. Die Freitext-Kommentare, in denen die Kunden ihre Gesamtzufriedenheit begründen, geben zusätzlich teils sehr konkrete Hinweise auf Optimierungspotenziale.

Einmal im Monat treffen sich die CX-Manager in Regelworkshops mit den Kundenlotsen und Touchpointverantwortlichen der jeweiligen Organisationseinheit zur allgemeinen Evaluation der aktuellen Zufriedenheitswerte. Des Weiteren findet einmal im Quartal ein Kundenreiseworkshop gemeinsam mit den CX-Managern, den Kundenlotsen und allen an der Customer Journey beteiligten Bereiche (u. a. Vertrieb, Call Center, Schadenzentren, Marketing, IT) statt, um in einer zwischenzeitlichen Prüfung der Ergebnisse hinsichtlich der Kundenzufriedenheit die umgesetzten Verbesserungsmaßnahmen zu analysieren, neue Handlungsfelder aufzudecken und Optimierungsmaßnahmen (weiter) zu entwickeln. Eine wichtige Arbeitshilfe sind dabei die jährlich von Market Research berechneten CX-Reports. Sie zeigen zusammenfassend, wie sich die Zufriedenheitswerte der Kunden (Ist-Werte) entlang der Customer Journey innerhalb eines Jahres sowie gegenüber dem Markt (Soll-Werte) entwickelt haben und, ob die definierten strategischen und operativen Kennzahlen des CX-Managements erreicht wurden.

Im nächsten Schritt erfolgt die Umsetzung der Verbesserungsmaßnahmen (‚Handeln‘; siehe Abb. 2). Die Maßnahmen können dabei sowohl eine Anpassung des Verhaltens (z. B. Zuverlässigkeit im Kundenkontakt) als auch prozessuale (z. B. ‚Welcome Package‘) und organisatorische Änderungen (z. B. Aufstockung der Telefonie Kapazitäten nach Unwettern mit vielen Schadenmeldungen) oder eine Kombination aus den Dreien sein. Beispielhaft wird hier kurz auf das sog. ‚Welcome Package‘ eingegangen, das als Touchpoint neu aufgenommen wurde, nachdem die Kunden in den Befragungen signalisiert haben, dass sie sich bei Erhalt der Police in einem dicken Paket mit viel Kleingedrucktem wie eine Nummer behandelt gefühlt haben. Des Weiteren ist das viele Kleingedruckte, das der Gesetzgeber fordert, für den Versicherungskunden wenig aufschlussreich für das Verständnis der abgeschlossenen Konditionen. *„Aus diesem Kundenfeedback heraus haben wir den neuen Touchpoint ‚Welcome Package‘ entwickelt: mit einer freundlichen, persönlichen Begrüßung, mit einer Übersicht aller Dienstleistungen auf einen Blick, um dem Kunden [das Verstehen der Versicherungsbedingungen zu erleichtern], die häufig auch aus rechtlichen Gründen sehr [komplex] formuliert sind“ (Exp_02)*. Die Kundenzufriedenheit mit den Informationen zum Vertragsabschluss ist dadurch deutlich gestiegen. Insgesamt ist mit Blick auf die Customer Journeys festzuhalten, dass diese einen lebenden Organismus darstellen, weshalb pro Jahr einige Touchpoints überflüssig oder neu gewünscht werden. Da sich die Relevanz der Touchpoints aus Kundensicht im Zeitverlauf regelmäßig verändert, ist es von enormer Bedeutung, die Kundenerwartungen und -wünsche kontinuierlich zu erfragen und in das tägliche Handeln einzubeziehen.

Nachdem der Closed Loop einmal durchlaufen wurde, werden auf Basis der laufenden Kundenzufriedenheitsbefragungen die Erfolge der Optimierungsmaßnahmen evaluiert (‚Prüfen‘; siehe Abb. 2), neue Handlungsfelder identifiziert und der Kreislauf der CX-Methodik beginnt erneut. Es wird ersichtlich, dass der Closed Loop der NÜRNBERGER Versicherung je Geschäftsbereich einen in sich geschlossenen Managementprozess innerhalb der Umsetzungsphase des CEM-Prozesses nach Mayer-Vorfelder (2012a) mit ähnlichen Teilphasen (Analyse, Planung, Umsetzung, Kontrolle) darstellt. Wie bereits in Abschn. 4.2 beschrieben, muss daher mit Blick auf die Umsetzungsphase zwischen der Umsetzung im Geschäftsbereich und der gesamtheitlichen Umsetzung des CX-Managements auf Konzernebene unterschieden werden (siehe Abb. 1).

*Einführung eines CRM-Systems*

Bei der NÜRNBERGER Versicherung sind in 2021 die Vorarbeiten für die Einführung eines Endkunden CRM-Systems angelaufen. Mit Blick auf das CX-Management ermöglicht das CRM-System einerseits personalisierte Befragungen, die aufgrund der Informationen aus dem CRM sowohl weniger (allgemeine) Fragen als auch auf den Geschäftsvorfall oder Touchpoint spezifisch abgestimmte Fragen enthalten. Andererseits besteht zudem auch die Möglichkeit, die Ergebnisse und Daten aus dem Customer Feedback System den Kennzahlensystemen anderer Unternehmensbereiche zur Verfügung zu stellen. Dies zeigt erneut die Bereitschaft des Unternehmens zur kontinuierlichen und vollumfänglichen Ausrichtung an den Kunden zur Verbesserung der Customer Experience an allen Touchpoints und damit zur Steigerung der Kundenzufriedenheit.

##### Schaffung einer kunden- und erlebnisorientierten Unternehmenskultur

Die Bedeutung, die die NÜRNBERGER Versicherung der Kundenzufriedenheit und -zentrierung zuschreibt, wird durch deren ganzheitliche Verankerung in der Unternehmenskultur deutlich. Die beiden Fokusthemen finden sich nicht nur in der Vision und Mission des Unternehmens, dessen Leitbild und in den Markenwerten wieder, sondern auch im Geschäfts- und im Nachhaltigkeitsbericht. Das CX-Management leistet als wichtigste Methode zur Steigerung der Kundenzufriedenheit einen bedeutenden Beitrag zu der Relevanz und dem Verständnis für Kundenerlebnisse im Unternehmen.

Das Einstellen der CX-Manager als strategische Treiber und die Benennung der Kundenlotsen und Touchpointverantwortlichen als operative Treiber der Kundenzentrierung stellen zentrale Elemente der Mobilisierung innerhalb der NÜRNBERGER Versicherung dar. Die CX-Manager tragen beispielsweise durch verschiedene Kommunikationsmaßnahmen und die Vorstellung des CX-Managements auf unternehmensinternen (Präsenz‑)Veranstaltungen als Multiplikatoren zur konzernweiten Aufklärung über die Fokusthemen bei. Die Kundenlotsen und Touchpointverantwortlichen können als Mediatoren gesehen werden, die die Themen des CX-Managements in die Fachbereiche tragen, dort die Aufmerksamkeit dafür steigern und die Umsetzung der Verbesserungsmaßnahmen vorantreiben.

Eine entscheidende Rolle für die Mobilisierung spielen zudem die Vorstände und Führungskräfte, die den Wandel hin zur Kundenzentrierung vorleben. Dies geschieht zum Beispiel durch das Format ‚Aus erster Hand‘, in dem die Vorstände direkt und transparent die Belegschaft über verschiedene Themen informieren. *Exp_03* und *Exp_04* bewerten die schnelle Schaffung von Strukturen und entsprechender Jobpositionen sowie die Aufgeschlossenheit der NÜRNBERGER Versicherung gegenüber entsprechenden Investitionen und auch das Mitziehen der Führungskräfte als sehr positiv.

Eine nicht zu unterschätzende Wirkung, die die Arbeit mit dem CX-Management weiterhin auf die Unternehmenskultur hat, ist die Steigerung der Mitarbeiterzufriedenheit. Diese resultiert zum einen aus positiven Kundenfeedbacks, die den Mitarbeitern im Kundenkontakt (der meist aufgrund tendenziell negativ assoziierter Gründe wie Problemen oder Beschwerden zustande kommt) große Wertschätzung für ihre tägliche Arbeit entgegenbringen. Des Weiteren eröffnet das CX-Management den Mitarbeitern die Möglichkeit, sich direkt im Unternehmen einzubringen und weiterzuentwickeln. Auch ein neu ermöglichter Entscheidungsspielraum im Sinne des Empowerments erhöht die Mitarbeiterzufriedenheit. *„Das alles sollte CX-Management bewirken. Die Mitarbeiter sollten gepackt werden und ein Teil davon sein wollen. Die Tätigkeit und Zusammenarbeit, die durch das CX-Management gefördert wird, führt zu mehr Motivation, Leistungsbereitschaft und Innovation bei den Mitarbeitern“ (Exp_04)*.

#### Erfolgskontrolle auf Konzernebene

Wie in Abschn. 4.2.2 bereits erwähnt, erfolgt die Erfolgskontrolle des CX-Managements sowohl auf Konzernebene als auch innerhalb des Closed Loops der CX-Methodik in der Arbeit mit den Ergebnissen der Kundenbefragungen. Unternehmen stellen sich im Zuge der (geplanten) Implementierung eines CEM derzeit sicherlich branchenübergreifend regelmäßig die entscheidende Frage nach dem potenziellen Erfolg und der Wirkung des CEM. Mit Blick auf die Entwicklung der Position der NÜRNBERGER Versicherung in den KUBUS Studien der vergangenen Jahre hat sich das Unternehmen seit dem Beginn des Aufbaus eines CX-Managements um zehn Plätze verbessert. Das Unternehmen liegt insgesamt noch unter den angestrebten Werten der Kundenzufriedenheit, ist dem gesteckten Konzernziel aber bereits deutlich nähergekommen. Betriebswirtschaftlich wirkt sich eine steigende Kundenzufriedenheit auf eine engere Kundenbindung und eine höhere Kundenloyalität aus. Dies führt wiederum zu höheren Wiederabschluss- und Cross-Selling-Quoten sowie weniger Stornos. Das Fazit von Seiten der NÜRNBERGER Versicherung lautet daher: CX-Management wirkt. In dem Wissen um die dynamischen Entwicklungen der Kundenzufriedenheit und zur finalen Erreichung des Konzernziels bedarf es nichtsdestotrotz der kontinuierlichen Optimierung sowie Weiterentwicklung des CX-Managements zur stetigen Verbesserung der Kundenerlebnisse und -zufriedenheiten.

### Ausblick

In der bisherigen Forschung wurde dem CEM sowie der ganzheitlichen Verbesserung der Customer Experience häufig eine bedeutende Rolle zur Erlangung eines Wettbewerbsvorteils (z. B. über eine gesteigerte Kundenzufriedenheit und -loyalität) zugeschrieben (u. a. Homburg et al. 2017; McColl-Kennedy et al. 2019; Verhoef et al. 2009). Mit Blick auf die bereits beschriebenen Marktgegebenheiten und -entwicklungen lässt sich dies aus Sicht der Experten für den aktuellen Versicherungsmarkt nicht eindeutig bestätigen. Grundsätzlich wird darauf verwiesen, dass sich alleine durch die Implementierung einer entsprechenden CEM Aufbauorganisation weder ein Wettbewerbsvorteil, noch ein Alleinstellungsmerkmal für ein Versicherungsunternehmen ergibt, da bereits viele Versicherer in Deutschland ein CEM aufgebaut und installiert haben.

*„Aber in dem Kreis der Versicherer, die bisher vermittlerorientiert waren und sind, kann das Customer Experience Management helfen, die Kundensicht […] besser mit einzubeziehen und das [bringt] einen Wettbewerbsvorteil“* bestätigt *Exp_01*. Um dies zu erreichen, bedarf es jedoch neben ausreichend Investitionen und Ressourceneinsatz vor allem Veränderungen auf vielerlei Ebenen. Neben der Entwicklung eines Programms, der Zuteilung entsprechender Zuständigkeiten und strukturellen Veränderungen ist die systematische und kontinuierliche Arbeit mit dem CEM zur Verbesserung der Kundenerlebnisse zentral:* „Ganz wichtig ist: Aus dem Messen muss ich in die Umsetzung kommen. CX-Management ist mehr als das Messen!“ (Exp_03)*. Zu guter Letzt muss der kulturelle Wandel hin zur Kundenzentrierung im gesamten Unternehmen von jedem einzelnen Mitarbeiter gelebt werden, denn CEM sollte *„nichts ‚on top‘ sein, sondern es muss in Fleisch und Blut übergehen!“ (Exp_04)*.

Mit Blick auf die Erlangung eines Alleinstellungsmerkmals durch die Arbeit mit einem CEM haben die Experten hinsichtlich des CX-Managements der NÜRNBERGER Versicherung unterschiedliche Meinungen. Einerseits zeigt sich auch hier in Zitaten wie beispielsweise *„dafür müssten ganz andere Aspekte geboten werden“ (Exp_09)* und *„Da sind glaube ich schon etliche Wettbewerber besser als wir“ (Exp_01)*, dass ein Teil der Versicherungswirtschaft bereits mit höherem Einsatz von Investitionen und Ressourcen an der Thematik arbeitet. Andere Experten sehen hingegen im Zusammenspiel aus dem CX-Management und der Marke, durch die entsprechende Erfüllung der Markenversprechen, eine Chance der NÜRNBERGER Versicherung zur Schaffung einer nachhaltigen Differenzierung.

Nach mehrheitlicher Meinung der Experten wird das CEM für die Versicherungsbranche zukünftig noch an Relevanz zunehmen. Aussagen wie *„Customer Experience Management ist gekommen um zu bleiben“ (Exp_04)* und *„Aus meiner Sicht wird dieses Thema Kundenservice und Customer Experience Management für die Versicherungsbranche State-of-the-Art werden müssen“ (Exp_02) *verdeutlichen, dass der Managementansatz aus der Versicherungsbranche nicht mehr wegzudenken ist. Die Experten sind überzeugt, dass das CEM in Zukunft *„ein fest installierter Bestandteil sein [wird], ohne den kein Versicherungsunternehmen auf dem Markt bestehen kann“ (Exp_06) *und, dass *„Unternehmen, die bei den [Customer Experience Management]-Aktivitäten nachlassen, […] das Nachsehen haben [werden], weil ihnen wesentliche Grundlagen zur Weiterentwicklung ihres Geschäftsmodells fehlen werden“ (Exp_03)*.

Auch, wenn dessen Stellenwert in der NÜRNBERGER Versicherung bereits sehr hoch ist (siehe Abschn. 4.2), bedarf es zur optimalen Umsetzung des CX-Managements auch der Schaffung einer darauf ausgelegten systemtechnischen Infrastruktur. Die größte Restriktion der vollumfänglichen Kundenzentrierung innerhalb der NÜRNBERGER Versicherung sind derzeit die technischen Systeme, die kundenorientierte Veränderungen behindern, obwohl Führungskräfte und Mitarbeiter die Handlungsfelder durch die Anwendung der CX-Methodik bereits kennen (z. B. in der Textgestaltung). Technologiewechsel setzen dabei die Grenzen des Machbaren. Weiterhin sind die Mobilisierung und Haltungsänderung aller Mitarbeiter, laut *Exp_03 *zwei wichtige Erfolgsfaktoren des CEM, sowie der Bekanntheitsgrad des CX-Managements als Team und Thema aufgrund der Neuartigkeit steigerungsfähig: *„Aber wie alles, was neu ist, dauert das wahrscheinlich ziemlich lang, bis alle Mitarbeiter und Führungskräfte die Notwendigkeit [des CX-Managements] so richtig verinnerlicht haben“ (Exp_01)*. Zukünftig sollen daher *„alle an der Customer Journey beteiligten Bereiche in der NÜRNBERGER [befähigt sein], CX-Themen selbstständig voranzubringen“ (Exp_05)*.

## Diskussion

Die vorliegende Studie gibt einen praxisnahen Einblick in das in Bezug auf die Versicherungsbranche noch wenig untersuchte Forschungsfeld des CEM. Wie bereits in Abschn. 4.2 erläutert, kann das von Mayer-Vorfelder (2012a) vorgeschlagene Konzept eines entscheidungsorientierten CEM nicht unverändert auf die Versicherungsbranche angewandt werden, diente aber im Kern aufgrund dessen Dienstleistungsfokus, der Praxisnähe und der umfassenden Betrachtungsweise des Managementansatzes als treffende Vergleichsbasis für die Analyse des CX-Managements der NÜRNBERGER Versicherung. Die Ergebnisse zeigen, dass das Unternehmen bereits in allen Teilbereichen des Managementprozesses tätig ist und eine Vielzahl unternehmensweiter Veränderungen hin zu einer ganzheitlichen Kundenzentrierung vorgenommen hat.

Homburg et al. (2017) haben eine Typologie entwickelt, die Unternehmen anhand deren Größe (≤ 250 Mitarbeiter vs. > 250 Mitarbeiter) und der Kontinuität der Austauschbeziehung im Rahmen des Kerngeschäfts (transaktionsorientiert vs. beziehungsorientiert) in vier Muster einordnet. Diese Muster unterscheiden sich hinsichtlich der Schwerpunkte in der Ausgestaltung des CEM in Bezug auf die kulturelle Orientierung, strategischen Richtungsvorgaben und unternehmerischen Fähigkeiten (Homburg et al. 2017). Die NÜRNBERGER Versicherung würde mit allein rund 3000 Mitarbeitern am Firmensitz Nürnberg und dem Fokus auf langfristige Kundenbeziehungen hinsichtlich ihres CX-Managements in das vierte Muster nach Homburg et al. (2017) fallen. Unternehmen in diesem Muster gewichten die drei Schwerpunkte in etwa gleich stark, wobei in Bezug auf die kulturelle Orientierung der Fokus auf unternehmensübergreifende Kooperationen liegt und hinsichtlich der strategischen Richtungsvorgaben die Konnektivität der Touchpoints hervorgehoben werden (Homburg et al. 2017). Obwohl die NÜRNBERGER Versicherung vereinzelte Kooperationen unterhält (z. B. Malteser-Schutzbrief, TeleClinic), wird dem Unternehmen hinsichtlich dessen kultureller Orientierung eher eine Touchpoint Journey-Orientierung zugeschrieben, da die Customer Journeys und die entsprechenden Touchpoints als Grundlage des CX-Managements fungieren. Des Weiteren richtet sich das Unternehmen strategisch vermehrt auf die Konsistenz und Kontext-Sensitivität von Touchpoints aus, wenngleich auch die Konnektivität der Touchpoints als strategische Richtungsvorgabe in das CX-Management einbezogen wird. Im Einklang mit Homburg et al. (2017) weist auch die NÜRNBERGER Versicherung alle vier unternehmerischen Fähigkeiten zur stetigen Erneuerung und Verbesserung der Customer Experience auf: die Nutzung des Customer Journey Mappings als Ausgangspunkt für die Optimierung der Customer Experience (Touchpoint Journey Design), die Touchpoint Priorisierung über Treiberanalysen, das Monitoring der Kundenzufriedenheit durch kontinuierliche Kundenbefragungen (Touchpoint Journey Monitoring) und die kontinuierliche Interpretation der Kundenfeedbacks und den weiteren Einbezug von Kunden (z. B. Kunden-Communities) in die Entwicklung sowie Modifikation der Touchpoints und Customer Journeys (Touchpoint Adaption).

Zusammenfassend lässt sich feststellen, dass die NÜRNBERGER Versicherung als Fallbeispiel für die Versicherungswirtschaft durch dessen unternehmensweite Umsetzung sowie kontinuierliche Arbeit des CX-Managements bereits erste betriebswirtschaftliche Erfolge erzielt hat. Dies zeigt, dass die dem Managementansatz aktuell aus wissenschaftlicher und praxisbezogener Perspektive zugeschriebene Relevanz auch für die Versicherungsbranche berechtigt ist. Wenn es einem Versicherungsunternehmen also gelingt, durch den gezielten Einsatz von Maßnahmen des CEM das Kundenerlebnis zu optimieren und die Kundenerwartungen zu erfüllen, kann es unter anderem mit einer gesteigerten Kunden- und Mitarbeiterzufriedenheit, geringeren Kündigungsquoten und höheren Umsätzen belohnt werden (u. a. auch Helkkula et al. 2012; Otto et al. 2020; Rawson et al. 2013; Zomerdijk und Voss 2010). CEM kann dann einen wertvollen Beitrag zum Erfolg von Versicherungsunternehmen leisten. Der Managementansatz ist daher nicht mehr aus der Versicherungswirtschaft wegzudenken.

Diese Studie trägt zur Untersuchung der (Re‑)Organisation von Unternehmen für ein erfolgreiches Management der Customer Experience bei (Lemon und Verhoef 2016). Nichtsdestotrotz weist die Studie Limitationen auf. Bisher besteht der Großteil der Forschung zu Customer Experience (Management) aus konzeptionellen Arbeiten, z. B. auf Basis von Fallstudien, Literaturüberblicken oder Tiefeninterviews (u. a. Becker und Jaakkola 2020; Homburg et al. 2017; Lemon und Verhoef 2016; Meyer und Schwager 2007). Die durchgeführten Experteninterviews und die Betrachtung eines expliziten Fallbeispiels reihen sich in die qualitative Forschungsmethodik ein. Weitere empirische Untersuchungen sollten die Wirkung und den Erfolg des CEM in der Versicherungswirtschaft quantitativ analysieren (Homburg et al. 2017). Erste vielversprechende Ergebnisse liefern Klink et al. (2021), die einen positiven Effekt der CEM Bemühungen eines Unternehmens auf die finanzielle Performance nachweisen.

Aufgrund der Befragung von zehn Experten eines Versicherungsunternehmens, müssen die Aussagen für die gesamte Versicherungswirtschaft entsprechend mit Vorsicht getroffen werden. In der Branche existieren, unter anderem mit Blick auf die Unterschiede zwischen Service- und Direktversicherer, verschiedene Ziele und Umsetzungsansätze des CEM. Eine breit angelegte Studie mit einer größeren Stichprobe sowie über unterschiedliche Versicherungsunternehmen hinweg könnte die Ergebnisse dieser Studie um weitere branchenspezifische Besonderheiten, strukturelle Unterschiede und systemseitige Aspekte hinsichtlich des Managementansatzes ergänzen. Es sei außerdem darauf hingewiesen, dass diese Studie im Sinne einer Momentaufnahme den Entwicklungsstand des CX-Managements der NÜRNBERGER Versicherung im Jahr 2020 darstellt. Die Durchführung einer Langzeitstudie könnte durch die Betrachtung desselben Fallbeispiels zu verschiedenen Zeitpunkten offenlegen, wie sich bestimmte Bedingungen im Laufe der Zeit verändern und welche verschiedenen Phasen der Implementierung und Weiterentwicklung des CEM in einem Unternehmen vollzogen werden (Jozić 2015; Yin 2009). Zudem wurde in dieser Studie der Vertrieb bewusst in den Hintergrund gerückt, um das CX-Management als übergeordneten und konzernweiten Managementansatz zur Steigerung der Kundenzentrierung und -zufriedenheit zu beleuchten. Nichtsdestotrotz wird dem Vertrieb insbesondere für Versicherungsunternehmen mit Vertriebspartnern seit jeher eine besondere Stellung im Hinblick auf die Customer Experience beigemessen. Im Vergleich zu Direktversicherern stellt dieser für Serviceversicherer einen entscheidenden Touchpoint in der Customer Journey dar und dessen Handlungsweisen haben damit einen wesentlichen Einfluss auf die Kundenzufriedenheit.

## Zusammenfassung und Schlussbemerkung

Versicherungsunternehmen stehen, wie viele (Dienstleistungs‑)Unternehmen, aufgrund einer Vielzahl von Veränderungen (u. a. verändertes Konsumentenverhalten, gestiegene Kundenerwartungen in Folge der Digitalisierung) vor der Herausforderung, ihren Kunden an jedem Touchpoint entlang der Customer Journey eine besondere sowie positive Customer Experience zu bieten. Dem CEM als Managementansatz wird dabei in der Versicherungsbranche eine entscheidende Rolle zugeschrieben. Dies zeigt sich unter anderem an der seit einige Jahren stark gestiegenen Anzahl an Versicherungsunternehmen, die an und mit einem CEM arbeiten. Jedoch wurde der Managementansatz für die Versicherungsbranche und ihre speziellen Marktgegebenheiten (z. B. erschwerte ganzheitliche Sicht auf den Kunden aufgrund der Spartentrennung; Theis und Wiener 2018) und -entwicklungen (z. B. ausgeschöpfte Differenzierungsmöglichkeiten auf Produkt- und Leistungsebene; Theis und Wiener 2018) bisher wenig erforscht.

Dieser Artikel beschäftigt sich daher mit dem Status quo des CEM und dessen Umsetzung in der Versicherungsbranche. Die Erkenntnisse der durchgeführten Experteninterviews gewähren praxisnahe Einblicke in den Aufbau, die konzernübergreifende Implementierung sowie die Ausgestaltung des CX-Managements der NÜRNBERGER Versicherung, die als Fallbeispiel für diese qualitative Studie herangezogen wurde. Der sehr hohe Stellenwert, den die NÜRNBERGER Versicherung dem CX-Management als auch der Kundenzentrierung zuweist, wird beispielweise durch die Schaffung einer gleichnamigen Organisationseinheit sowie deren Ansiedlung als Treiberfunktion im Bereich ‚Kundenbeziehungsmanagement‘ des neu geschaffenen Vorstandsressort ‚Kundenbeziehungsmanagement und Operations‘ ersichtlich. Häufig verändern oder schaffen Unternehmen erst langsam entsprechende Strukturen oder Funktionen, obwohl dies einen wichtigen ersten Schritt des Aufbaus eines CEM darstellt und ein positives Signal des Kulturwandels in das Unternehmen sendet.

Die Ergebnisse der Experteninterviews zeigen außerdem auf, dass der Managementprozess des CEM nach Mayer-Vorfelder (2012a), der als konzeptioneller Rahmen dieses Artikels dient, in der NÜRNBERGER Versicherung auf mehreren Ebenen sowie parallel abläuft anstatt einmalig und geradlinig (siehe Abb. 1). Auf Konzernebene erfolgen dabei übergreifende Aktivitäten der Planungsphase (u. a. CX-Management als Strategie-Baustein mit Ziel der Steigerung der Kundenzufriedenheit), der Umsetzungsphase (u. a. Schaffung geeigneter Unternehmensstrukturen und -kultur, Einführung neuer und Anpassung bestehender (IT-)Systeme) und der Kontrollphase (u. a. Gesamtzufriedenheit im Marktvergleich). Der Closed Loop der CX-Methodik (siehe Abb. 2), der auf Ebene der einzelnen Geschäftsbereiche parallel abläuft, bildet den Kernprozess der Umsetzung und Linienarbeit des CX-Managements der NÜRNBERGER Versicherung. Nachdem in einem Geschäftsbereich zunächst die jeweiligen Customer Journeys entwickelt wurden, dienen passgenaue und kontinuierlich durchgeführte Kundenbefragungen der Generierung von Wissen über die Kundenerwartungen und -bewertungen. Die Kundenbewertungen und deren Kundenzufriedenheitswerte bilden die Grundlage aller Maßnahmen zur Optimierung der Customer Experience. Die aus der täglichen Arbeit mit dem CX-Mess-System, aus verschiedenen Workshops sowie anhand der CX-Reports abgeleiteten Verbesserungsmaßnahmen reichen dabei von einer Anpassung des Verhaltens über prozessuale bis hin zu organisatorischen Änderungen. Auch eine Kombination aus den Dreien ist möglich. Anschließend erfolgt im Rahmen der laufenden Kundenzufriedenheitsbefragungen die Erfolgsmessung der Optimierungsmaßnahmen, auf Basis derer neue Handlungsfelder identifiziert werden können und der Closed Loop von vorne beginnt. Insgesamt lässt sich festhalten, dass die NÜRNBERGER Versicherung durch die unternehmensweite Umsetzung sowie kontinuierliche Arbeit des CX-Managements bereits erste Erfolge erzielt hat (u. a. Verbesserung der Kundenzufriedenheit und damit der Position in den KUBUS Studien).

Seit Jahren steigt die Kundenzufriedenheit sowie Weiterempfehlungsbereitschaft der Versicherungskunden, sodass die KUBUS Studie Privatkunden 2021 der Versicherungsbranche zuletzt ein neues Höchstniveau der Kundenzufriedenheit attestierte (MSR Consulting Group GmbH 2022). Auf dem stark umkämpften Markt ist es für den Unternehmenserfolg folglich wichtiger denn je, ein hohes Niveau an Kundenzufriedenheit zu generieren bzw. zu halten. Daher erscheint die Implementierung sowie kontinuierliche Optimierung bzw. Weiterentwicklung eines CEM für Versicherungsunternehmen in Zukunft als unerlässlich, um die Kundenzentrierung im gesamten Unternehmen zu fördern und zu einer stetigen Verbesserung der eigenen Kundenerlebnisse und -zufriedenheiten beizutragen.

## Footnotes

1 Nachfolgend wird bei explizitem Bezug auf das Customer Experience Management der NÜRNBERGER Versicherung der im Unternehmen etablierte Begriff CX-Management verwendet.

2 Aufgrund der COVID-19-Pandemie wurden alle Experteninterviews über das Tool MS Teams durchgeführt. Die Einzelinterviews wurden aus datenschutzrechtlichen Gründen nicht aufgezeichnet.

3 KUBUS (**Ku**ndenorientiertes **B**enchmarking zur **U**nternehmens**s**teuerung) Privatkunden ist eine bundesweite, jährliche Befragung der Kunden von 23 Versicherungs- und Finanzdienstleistungsunternehmen.

4 Eine Ausnahme stellt hierbei der Markenwert ‚unabhängig‘ dar, der sich auf die finanzielle Unabhängigkeit der NÜRNBERGER Versicherung bezieht.

## References

[CR1] Becker L, Jaakkola E (2020). Customer experience: fundamental premises and implications for research. J. Acad. Mark. Sci..

[CR2] Berry LL, Wall EA, Carbone LP (2006). Service clues and customer assessment of the service experience: lessons from marketing. Acad. Manag. Perspect..

[CR3] Bogner A, Littig B, Menz W (2014). Interviews mit Experten – Eine praxisorientierte Einführung.

[CR4] Brakus JJ, Schmitt BH, Zarantonello L (2009). Brand experience: What is it? How is it measured? Does it affect loyalty?. J. Mark..

[CR5] Bruhn M, Hadwich K (2012). Customer Experience.

[CR6] Bruhn M, Hadwich K, Zerres C (2021). Besonderheiten des Controlling im Dienstleistungsmarketing. Handbuch Marketing-Controlling.

[CR7] Büschken J, Otter T, Allenby GM (2013). The dimensionality of customer satisfaction survey responses and implications for driver analysis. Mark. Sci..

[CR8] Carlzon J (1987). Moments of Truth.

[CR9] Cepeda G, Martin D (2005). A review of case studies publishing in Management Decision 2003–2004. Guides and criteria for achieving quality in qualitative research. Manag. Decis..

[CR10] Döring N, Bortz J (2016). Forschungsmethoden und Evaluation in den Sozial- und Humanwissenschaften.

[CR11] Dyer WG, Wilkins AL (1991). Better stories, not better constructs, to generate better theory: a rejoinder to Eisenhardt. Acad. Manag. Rev..

[CR12] Edvardsson B, Enquist B, Johnston R (2005). Cocreating customer value through hyperreality in the prepurchase service experience. J. Serv. Res..

[CR13] Gao L, Melero-Polo I, Sese FJ (2020). Customer equity drivers, customer experience quality, and customer profitability in banking services: the moderating role of social influence. J. Serv. Res..

[CR14] Gentile C, Spiller N, Noci G (2007). How to sustain the customer experience: an overview of experience components that co-create value with the customer. Eur. Manag. J..

[CR15] Gilovich T, Gallo I (2020). Consumers’ pursuit of material and experiential purchases: a review. Consum. Psychol. Rev..

[CR16] Gläser J, Laudel G (2010). Experteninterviews und qualitative Inhaltsanalyse als Instrumente rekonstruierender Untersuchungen.

[CR17] Grewal D, Levy M, Kumar V (2009). Customer experience management in retailing: an organizing framework. J. Retail..

[CR18] Helkkula A, Kelleher C, Pihlström M (2012). Characterizing value as an experience: implications for service researchers and managers. J. Serv. Res..

[CR19] Holbrook MB, Hirschmann EC (1982). The experiential aspects of consumption: consumer fantasies, feelings, and fun. J. Consum. Res..

[CR20] Homburg C, Jozić D, Kuehnl C (2017). Customer experience management: toward implementing an evolving marketing concept. J. Acad. Mark. Sci..

[CR21] Jozić D (2015). Customer Experience Management – Eine empirische Analyse der Gestaltungsmöglichkeiten und Erfolgsauswirkungen.

[CR22] Klaus P, Maklan S (2012). EXQ: a multiple-item scale for assessing service experience. J. Serv. Manag..

[CR23] Klink RR, Zhang JQ, Athaide GA (2021). Measuring customer experience management and its impact on financial performance. Eur. J. Mark..

[CR24] Kreutzer RT, Rusnjak A, Schallmo DRA (2018). Customer Experience Management – wie man Kunden begeistern kann. Customer Experience im Zeitalter des Kunden.

[CR25] Kuehnl C, Jozic D, Homburg C (2019). Effective customer journey design: consumers’ conception, measurement, and consequences. J. Acad. Mark. Sci..

[CR26] Lemon KN, Verhoef PC (2016). Understanding customer experience throughout the customer journey. J. Mark..

[CR27] Mayer-Vorfelder M, Bruhn M, Hadwich K (2012). Customer Experience Management im Dienstleistungsbereich – Konzeption eines entscheidungsorientierten Managementansatzes. Customer Experience.

[CR28] Mayer-Vorfelder M (2012). Kundenerfahrungen im Dienstleistungsprozess – Eine theoretische und empirische Analyse.

[CR29] McColl-Kennedy JR, Zaki M, Lemon KN, Urmetzer F, Neely A (2019). Gaining customer experience insights that matter. J. Serv. Res..

[CR30] Meyer C, Schwager A (2007). Understanding customer experience. Harv. Bus. Rev..

[CR31] Moran G, Muzellec L, Nolan E (2014). Consumer moments of truth in the digital context: How “search” and “E-word of mouth” can fuel consumer decision making. J. Advert. Res..

[CR32] MSR Consulting Group GmbH: Kundenzufriedenheit der deutschen Assekuranz steigt auf ein neues Höchstniveau (2022). https://www.msr.de/presse/pressemitteilungen/kundenzufriedenheit-steigt-auf-hoechstniveau/, Zugegriffen: 21. Aug. 2022

[CR33] Otto AS, Szymanski DM, Varadarajan R (2020). Customer satisfaction and firm performance: insights from over a quarter century of empirical research. J. Acad. Mark. Sci..

[CR34] Palmer A (2010). Customer experience management: a critical review of an emerging idea. J. Serv. Mark..

[CR35] Parasuraman A, Zeithaml VA, Berry LL (1985). A conceptual model of service quality and its implications for future research. J. Mark..

[CR36] Patrício L, Fisk RP, Falcão e Cunha J, Constantine L (2011). Multilevel service design: from customer value constellation to service experience blueprinting. J. Serv. Res..

[CR37] Pine BJI, Gilmore JH (1998). Welcome to the experience economy. Harv. Bus. Rev..

[CR38] Prahalad CK, Ramaswamy V (2003). The new frontier of experience innovation. MIT Sloan Manage. Rev..

[CR39] Puccinelli NM, Goodstein RC, Grewal D, Price R, Raghubir P, Stewart D (2009). Customer experience management in retailing: understanding the buying process. J. Retail..

[CR40] Rawson A, Duncan E, Jones C (2013). The truth about customer experience. Harv. Bus. Rev..

[CR41] Roethlisberger FJ (1977). The Elusive Phenomena: An Autobiographical Account of My Work in the Field of Organizational Behavior at the Harvard Business School.

[CR42] Rosenbaum MS, Otalora ML, Ramírez GC (2017). How to create a realistic customer journey map. Bus. Horiz..

[CR43] Schmitt B (2003). Customer Experience Management: A Revolutionary Approach to Connecting with Your Customer.

[CR44] Sirius Campus (2020). Multi-Channel-Management in Corona Zeiten: Neue Digitalkompetenz und Kontaktpräferenzen von Versicherungskunden.

[CR45] Steffen A, Doppler S (2019). Einführung in die Qualitative Marktforschung: Design – Datengewinnung – Datenauswertung.

[CR46] Stoeckli E, Dremel C, Uebernickel F (2018). Exploring characteristics and transformational capabilities of InsurTech innovations to understand insurance value creation in a digital world. Electron. Mark..

[CR47] Teixeira J, Patrício L, Nunes NJ, Nóbrega L, Fisk RP, Constantine L (2012). Customer experience modeling: From customer experience to service design. J. Serv. Manag..

[CR48] Theis A, Wiener K (2018). Anbieterlandschaft am Versicherungsmarkt: Ein Ausblick.

[CR49] Van Boven L, Gilovich T (2003). To do or to have? That is the question. J. Pers. Soc. Psychol..

[CR50] Verhoef PC, Lemon KN, Parasuraman A, Roggeveen A, Tsiros M, Schlesinger LA (2009). Customer experience creation: determinants, dynamics and management strategies. J. Retail..

[CR51] Yin RK (2009). Case Study Research: Design and Methods.

[CR52] Zeithaml VA, Donnelly JH, George WR (1981). How consumer evaluation processes differ between goods and services. Marketing of Services.

[CR53] Zomerdijk LG, Voss CA (2010). Service design for experience-centric services. J. Serv. Res..

